# Design and characterisation of piperazine-benzofuran integrated dinitrobenzenesulfonamide as *Mycobacterium tuberculosis* H37Rv strain inhibitors

**DOI:** 10.1080/14756366.2021.1956914

**Published:** 2021-07-29

**Authors:** Vallabhaneni S. Murthy, Yasinalli Tamboli, Vagolu Siva Krishna, Dharmarajan Sriram, Siddique Akber Ansari, Abdullah A. Alarfaj, Abdurahman H. Hirad, Vijayaparthasarathi Vijayakumar

**Affiliations:** a Centre for Organic and Medicinal Chemistry, Department of Chemistry, School of Advanced Sciences, VIT University, Vellore, India; b Medicinal Chemistry and Antimycobacterial Research Laboratory, Pharmacy Group, Birla Institute of Technology and Science, Pilani, Hyderabad Campus, Hyderabad, India; c Department of Pharmaceutical Chemistry, College of Pharmacy, King Saud University, Riyadh, Saudi Arabia; d Department of Botany and Microbiology, College of Science, King Saud University, Riyadh, Saudi Arabia

**Keywords:** Piperazine-benzofuran, 2,4-dinitrobenzene sulphonamide, amino acid, hybridisation, anti-TB

## Abstract

Molecular hybridisation of four bioactive fragments piperazine, substituted-benzofuran, amino acids, and 2,4-dinitrobenzenesulfonamide as single molecular architecture was designed. A series of new hybrids were synthesised and subjected to evaluation for their inhibitory activity against *Mycobacterium tuberculosis* (*Mtb*) H37Rv. **4d**–**f** and **4o** found to exhibit MIC as 1.56 µg/mL, equally active as ethambutol whereas **4a, 4c, 4j** displayed MIC 0.78 µg/mL were superior to ethambutol. Tested compounds demonstrated an excellent safety profile with very low toxicity, good selectivity index, and antioxidant properties. All the newly synthesised compounds were thoroughly characterised by analytical methods. The result was further supported by molecular modelling studies on the crystal structure of *Mycobacterium tuberculosis* enoyl reductase.

## Graphical Abstract

**Figure:**
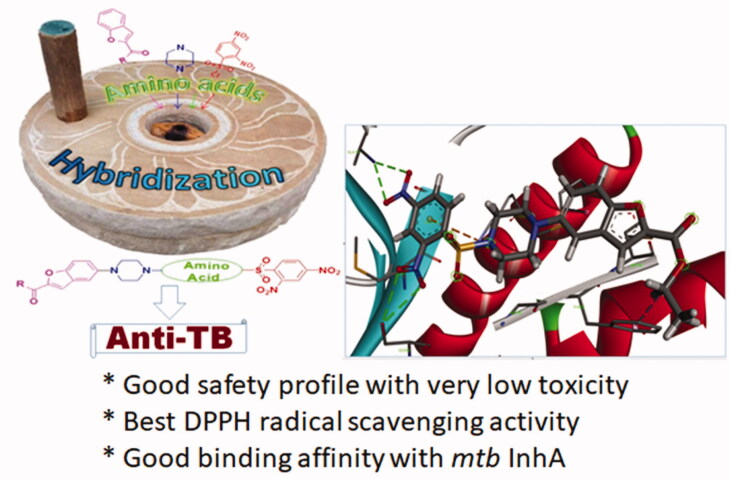

## 1. Introduction

Worldwide, tuberculosis (TB) is the leading cause from a single infectious agent with one of the top 10 causes of death. The WHO End-TB strategy aims to reduce TB deaths by 95% and to cut new cases by 90% between 2015 and 2035 with the ultimate aim to end the global TB epidemic^1^. Increased occurrence of multidrug and extensively drug-resistant TB constitutes an unresolved problem for extensive research regarding new anti TB drugs^2^.

Benzofuran is a class of fused heterocyclic compound having oxygen with a large spectrum of biological activities^3^^,^^4^ with relatively few examples of anti TB activity. The literature survey showed that piperazine incorporated benzofurans are important class of compounds, wherein piperazine expected to enhance selectivity and biological activity particularly in case of anti-TB activity^5^^,^^6^. 2-Substituted benzofurans exhibit promising anti-TB activity^7^. Amino acids were extensively utilised in molecular modification tools for the design and development of potential pharmaceutical drugs^8^.

The installation of a sulphonyl group in drug design has major advantages. It decreases hydrophobicity which may increase solubility in physiological conditions and subsequently could have an important impact on bioavailability. It has been observed that the sulphonyl group is an integral part of FDA-approved drugs including sulfamethoxazole to treat mycobacterial infections^9^^,^^10^. Nitro aryl sulphonamide derivatives enhance the lipophilicity of drug molecules, which play an important role in biological activities^11^. Indeed, sulphonamide analogues are known to exhibit a wide range of pharmacological activities particularly responsible for the enhanced anti-TB activity^12^^,^^13^. Moreover, our recent finding reveals that dinitro-substituted benzene derivatives have superior *in vitro* anti-TB activity^6^ compared with monosubstituted benzene derivatives probably due to electron-deficient aromaticity^14^.

**Figure 1.**
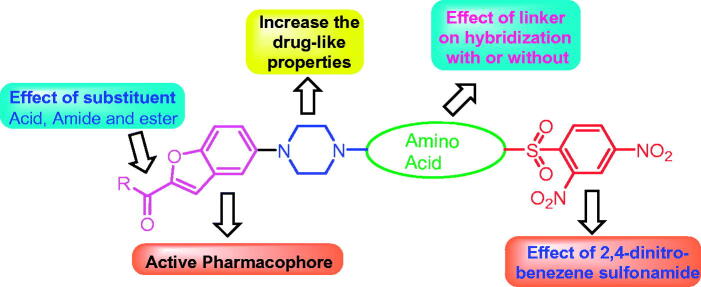
Molecular hybridisation of designed molecules.

The combination of pharmacophoric moieties of different bioactive compounds to produce a new hybrid with improved affinity, efficacy is a well-known concept in drug design and development^15^. Based on our prior efforts for the development of novel anti-TB agents, the aim of this study was to design new hybrid derivatives with increased activity against *Mycobacterium tuberculosis* (*Mtb*)^6^. Single hybrid architecture was successfully designed by linking four bioactive components such as substituted piperazine, 2-benzofuran, amino acids, and 2,4-dinitro-benzenesulfonamide. A more hydrophobic benzofuran nucleus was utilised to enhance the hydrophobicity of the hybrid. It was decided to retain 2,4-dinitrobenzene sulphonamide scaffold in the further structure–activity exploration. The structural diversity was achieved by modification at 2-benzofuran as well as linking with diverse amino acids as shown in Figure 1.

## 2. Results and discussion

### 2.1. Chemistry

**Scheme 1.**
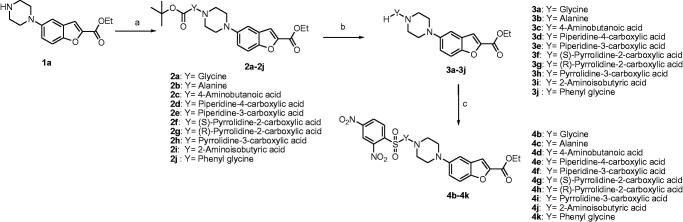
Synthesis of **4b**–**k**. Reagents: (a) HATU, DIPEA, DMF; (b) TFA, DCM; (c) 2,4-dinitrobenzenesulfonyl chloride, DIPEA.

**Scheme 2.**
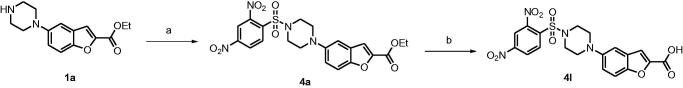
Synthesis of **4a** and **4l**. Reagents: (a) 2,4-dinitrobenzenesulfonyl chloride, DIPEA, DCM; (b**)** LiOH.H_2_O, THF, water, ethanol.

**Scheme 3.**
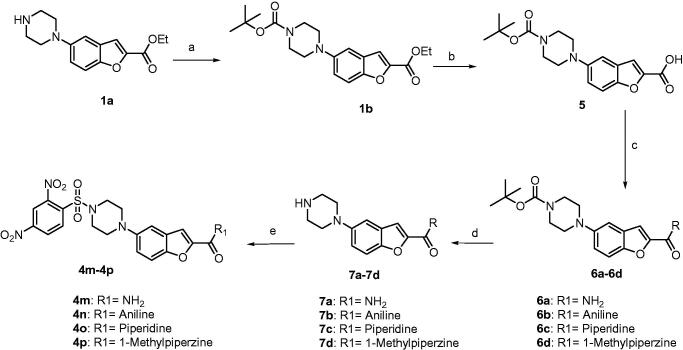
Synthesis of **4m**–**p**. Reagents: (a) Boc-anhydride, DIPEA, DCM; (b) LiOH, THF, water, ethanol; (c) HATU, DIPEA, DMF; (d) TFA, DCM; (e) 2,4-dinitrobenzenesulfonyl chloride, DIPEA, DCM.

The synthesis of 2,4-dinitrobenzenesulfonamide derivatives **4b**–**k** is described in Scheme 1. Amide derivatives **2a**–**j** were synthesised by a coupling reaction between ethyl 5-(piperazin-1-yl) benzofuran-2-carboxylate^4^^,^^5^ (**1a**) and Boc protected amino acids using HATU as a coupling reagent and DIPEA as a base. Further, the Boc group was deprotected using trifluoroacetic acid to give corresponding amine derivatives **3a**–**j**. Finally, commercially available 2,4-dinitrobenzene sulphonyl chloride is treated with amines **3a**–**j** in presence of a base DIPEA to afford the compounds **4b**–**k**. Ethyl 5-(piperazin-1-yl)benzofuran-2-carboxylate (**1a)** was treated with 2,4-dinitro benzenesulfonyl chloride to afford **4a**, which in turn is subjected to the hydrolysis using lithium hydroxide to afford the corresponding acid derivative **4l**. Unfortunately, attempts for purification of **4l** failed (shown in Scheme 2). Boc protection of **1a** leads to **1b** which upon basic hydrolysis uses lithium hydroxide to afford acid derivative **5**. Compound **5** was treated with the Boc protected amino acids with HATU and DIPEA to afford corresponding amide derivatives **6a**–**d**. The Boc deprotection by TFA of **6a**–**d** affords the corresponding amine derivatives **7a**–**d**. 2,4-Dinitrobenzenesulfonyl chloride was treated with amines **7a**–**d** to afford the compounds **4m**–**p** as described in Scheme 3. All the newly synthesised compounds were purified by column chromatography using 100–200 mesh silica gel with 2–6% methanol in dichloromethane as eluent, followed by triturating with n-pentane or diethyl ether. All the synthesised compounds were confirmed by analytical and spectral data (^1^H NMR, ^13^C NMR, LCMS, and elemental analysis).

### 2.2. Anti-TB activity

All the newly synthesised hybrid compounds **4a**–**p** was screened for their *in vitro* anti-TB activity against *Mtb* H37Rv (ATCC27294) using agar dilution method and their minimum inhibitory concentration (MIC) value has been determined by averaging of the triplicates. The preliminary MIC values (µg/mL) of **4a**–**p** along with the standard drugs for comparison are furnished in Table 1. These hybrids screened and compared to first-line anti-TB drugs such as isoniazid (0.05 µg/mL), rifampicin (0.1 µg/mL), and ethambutol (1.56 µg/mL). All the compounds have exhibited *in vitro* activity against *Mtb* with MIC ranging from 0.78 to >25 µg/mL. Among all these hybrids, **4d**–**f** and **4o** were found to exhibit MIC as 1.56 µg/mL, equally potent as ethambutol whereas **4a, 4c, 4j** displayed MIC 0.78 µg/mL which were superior to ethambutol and inferior with respective to isoniazid and rifampicin. Acid derivative **4b** and amide derivatives **4m**–**n, 4p** were found to be less potent than ethyl ester derivative **4a**. Moderate decline in activity was observed with amino acid conjugation between piperidine and dinitrosulfonamide except for alanine and 2-aminoisobutyric acid compared with **4a**.

**Table 1.** Anti-TB activity, cytotoxicity, selective index, and DPPH radical scavenging activities of compound **4a**–**p** (μg/mL).

Compound	Anti-TB (MIC) (μg/mL)	% Cell inhibition at 50 μg/mL	Human cell lines A549		DPPH IC_50_ (μg/mL)
			IC_50_ approximation (μg/mL)	SI index	
**4a**	0.78 ± 0.36	20.80	>50	>60	15.25
**4b**	3.12 ± 1.47	37.41	>50	>15	50.33
**4c**	0.78 ± 0.18	30.46	>50	>60	13.39
**4d**	1.56 ± 0.36	35.95	>50	>30	33.50
**4e**	1.56 ± 0.97	18.95	>50	>30	45.21
**4f**	1.56 ± 0.73	22.75	>50	>30	39.01
**4g**	6.25	29.64	>50	<10	55.25
**4h**	>25	33.08	>50	<10	49.75
**4i**	6.25	19.38	>50	<10	52.44
**4j**	0.78 ± 0.48	39.28	>50	>60	20.43
**4k**	>25	22.55	>50	<10	45.87
**4m**	25	23.65	>50	<10	45.44
**4n**	3.12 ± 1.10	27.98	>50	>15	40.64
**4o**	1.56 ± 0.36	31.58	>50	>30	19.27
**4p**	6.25	30.18	>50	<10	48.66
**Isoniazid**	0.05 ± 0.02	–	–	–	–
**Rifampicin**	0.10 ± 0.04	–	–	–	–
**Ethambutol**	1.56 ± 0.76	–	–	–	–
**Ascorbic acid**	–	–	–	–	12.7

Only most active compounds (MIC less than 3 mg/mL) were tested in triplicates.

### 2.3. Cytotoxicity studies

The safety profile of the active hybrids was also accessed by testing *in vitro* cytotoxicity against human cell-line A549 cells at 50 μg/mL concentration by (4,5-dimethylthiazol-2-yl)-2,5-diphenyltetrazolium bromide (MTT) assay. Percentage inhibitions of cells are reported in Table 1.

Most of the tested compound demonstrated a good safety profile with very low toxicity towards the A549 cells and showed a good selectivity index (IC_50_/MIC) except **4g**–**m** indicating the suitability of these compounds for further drug development.

### 2.4. Antioxidant activity

In TB, oxidative stress may result in tissue inflammation due to anti-TB drugs^16^. The synthesised compounds have shown promising anti-TB activity and have the potential to develop as lead compounds. Therefore, it is necessary to evaluate the synthesised compounds for their antioxidant activity. Antioxidant activities of the synthesised compounds were measured using 2,2-diphenyl-1-picrylhydrazyl (DPPH) radical scavenging assay^17^. DPPH radical scavenging activity is the most commonly used method for screening the antioxidant activities of the various natural as well as synthetic antioxidants. A lower IC_50_ value indicates greater antioxidant activity. Compounds **4c** (IC_50_=13.39 µg/mL), **4a** (IC_50_=15.25 µg/mL) show good antioxidant activity compared to the standard antioxidant drug ascorbic acid (IC_50_=12.7 µg/mL). Among tested hybrids, compound **4c** showed the best DPPH radical scavenging activity, while compound **4g** showed the lowest activity when compared with standards (Table 1).

### 2.5. Molecular docking study

Molecular docking was utilised to ascertain the mode of action of synthesised derivatives. Enoyl-ACP reductase of the type II fatty acid synthase (FAS-II) system is an important enzyme in the mycobacteria which is involved in the biosynthesis of the mycolic acid, major constituents of the *Mtb* cell wall. Destruction of the *Mtb* cell wall via inhibition of the mycolic acid synthesis is an attractive strategy for the development of potent anti-mycobacterial agents. Due to the conservative nature of the InhA, it was considered to be the safe biological target, and targeting this enzyme may lead to potent and selective anti-mycobacterial agents.

**Figure 2.**
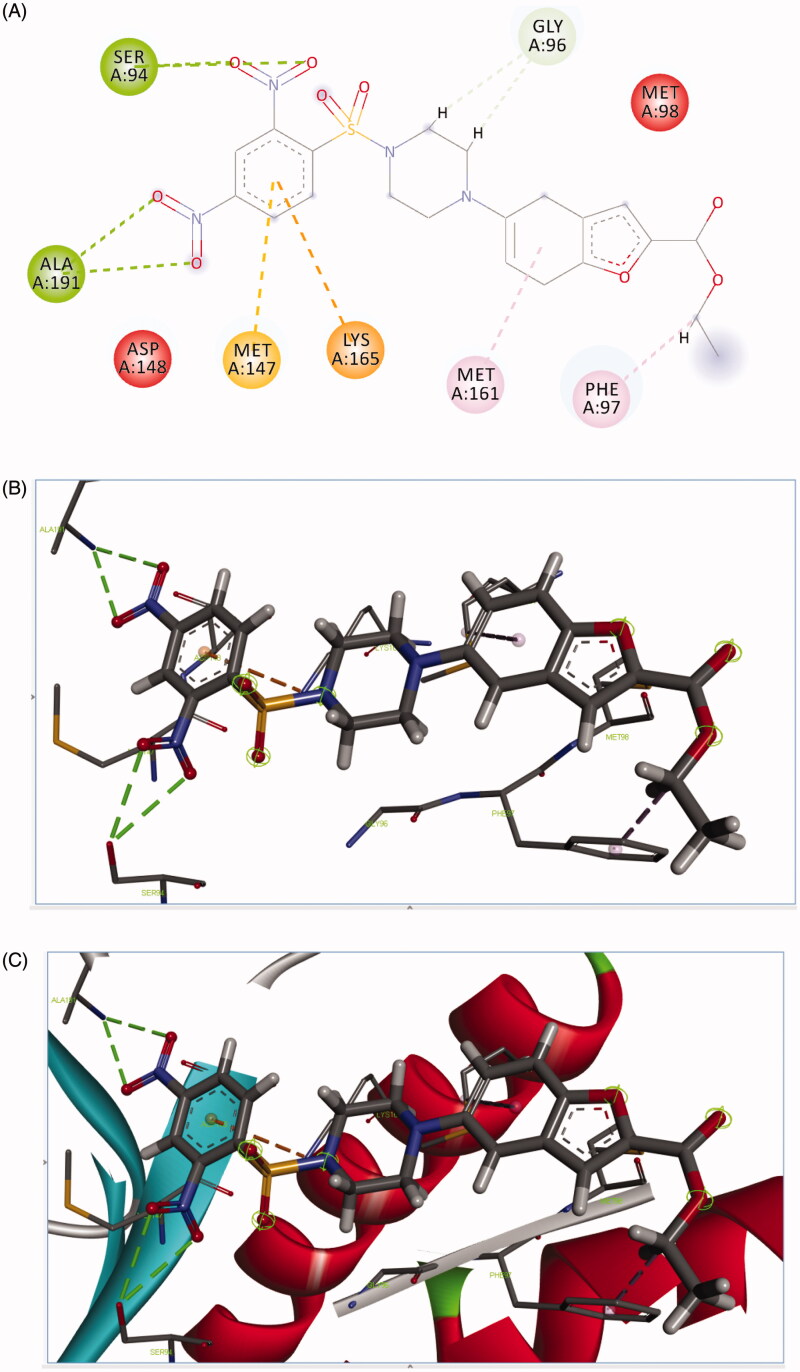
Binding mode of **4a** predicted by molecular docking.

All the synthesised derivatives have a good binding affinity with *Mtb* InhA which is indicated by their docking score ranging from −55.89 to −31.03. Most active derivative **4a** was found to be interacting with *Mtb* InhA via formation of hydrogen bond interaction with ALA191, SER 94 and carbon–hydrogen bond GLY 96 and Pi carbon LYS165 and Pi sulphur interaction MET 147, Pi alkyl with PHE 97 as shown in Figure 2(A–C).

**Figure 3.**
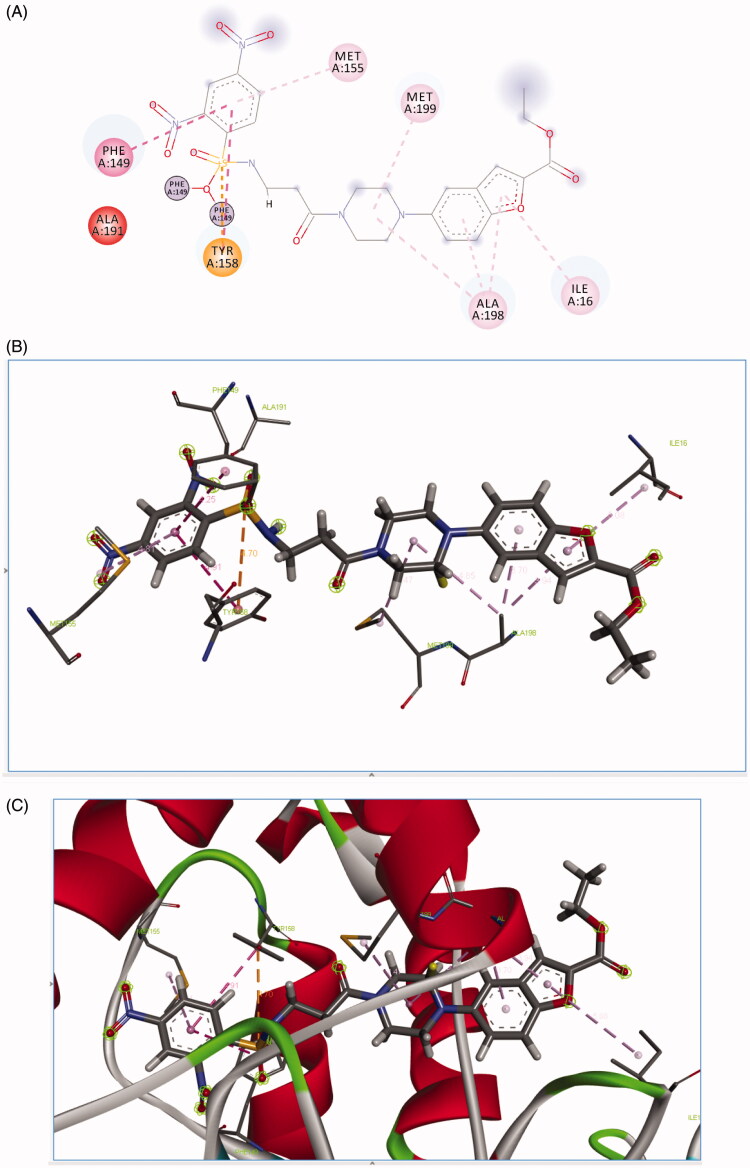
Binding mode of **4c** predicted by molecular docking.

**4c** was found to be interacting with *Mtb* InhA via formation of pi cation interaction with TYR 158, and PI–PI bond interaction with PHE149 as shown in Figure 3(A–C).

**Figure 4.**
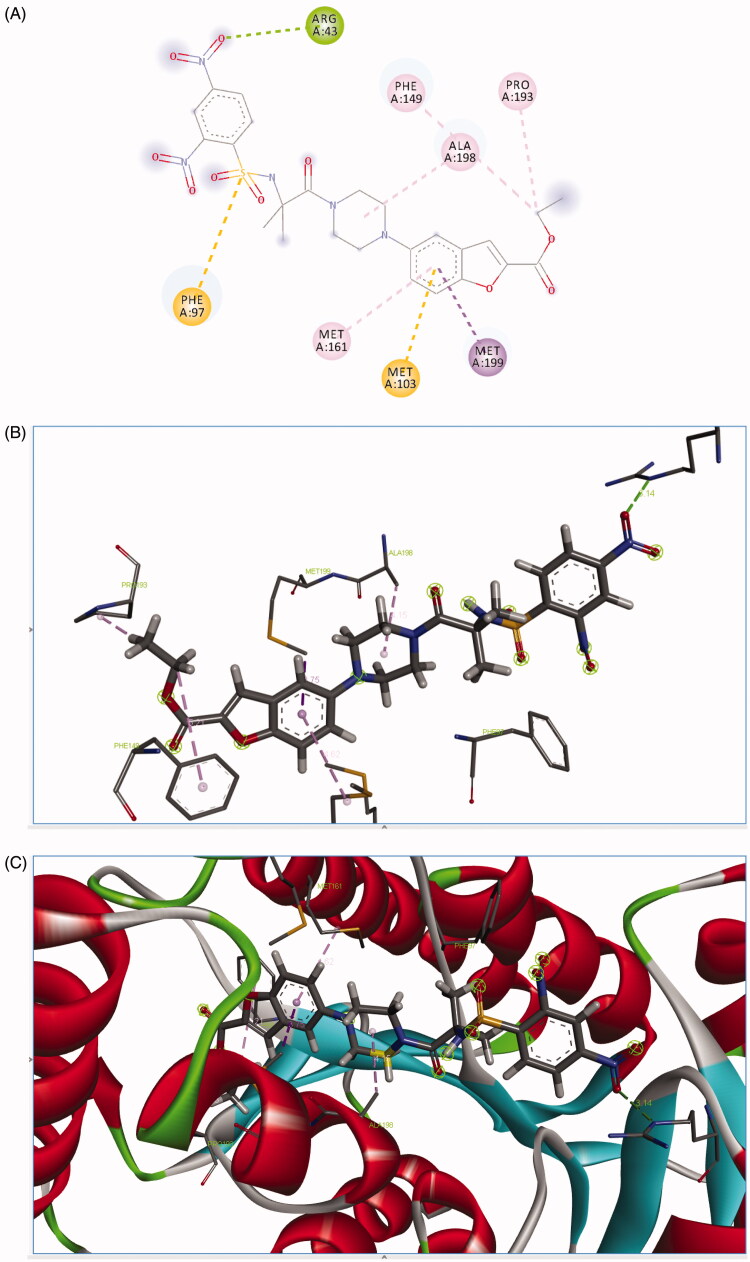
Binding mode of **4j** predicted by molecular docking.

**4j** was found to be interacting with *Mtb* InhA via the formation of hydrogen bond interaction with ARG43 and carbon–hydrogen bond GLY 96 and Pi sigma with MET 199 and Pi sulphur interaction PHE 97, MET103, Pi alkyl with PHE 149, ALA 198, PRO193 as shown in Figure 4(A–C).

## 3. Experimental

All reagents and solvents were purchased from commercial sources without further purification.

### 3.1. General procedure for the synthesis of 4a–4k and 4m–4p

To a solution of appropriate piperazine-benzofuran derivatives (1 equiv.) in DCM (10 vol), DIPEA (3 equiv.) and 2,4-dinitrobenzene-1-sulphonyl chloride (1.1 equiv.) were added and stirred at 25 °C for 2 h. TLC showed the completion of starting material and formation of the non-polar spot. The reaction mixture was concentrated to dryness and purified by silica gel (100–200 mesh) column chromatography using ethyl acetate in hexane as eluent to give corresponding sulphonamide derivatives **4a**–**4k** and **4m**–**4p**.

#### 3.1.1. Preparation of ethyl 5-(4-(2,4-dintrobenzenesulfonyl) piperazin-1-yl)benzofuran-2-carboxylate (4a)

Using 500 mg of ethyl 5-(piperazin-1-yl)benzofuran-3-carboxylate **1a** to get 360 mg, yield 32% as pale yellow solid. ^1^H NMR (400 MHz, DMSO-d_6_) *δ* 1.32 (t, *J* = 7.2 Hz, 3H, CH_3_), 3.24 (br s, 4H, 2x piperazine-CH_2_), 3.42 (br s, 4H, 2x piperazine-CH_2_), 4.34 (q, *J* = 7.2 Hz, 2H, CH_2_), 7.21 (s, 1H, Ar-H), 7.27 (dd, *J* = 9.2 Hz, 2.4, 1H, Ar-H), 7.59 (d, 1H, *J* = 9.2 Hz, Ar-H), 7.62 (s, 1H, Ar-H), 8.32 (d, *J* = 9.2 Hz, Ar-H), 8.60 (dd, *J* = 8.8 Hz, 2.4 Hz, 1H, Ar-H), 9.02 (s, 1H, Ar-H); ^13^C NMR (400 MHz, DMSO-d_6_): 14.10, 45.74, 49.62, 61.09, 108.64, 112.43, 114.07, 120.03, 120.32, 126.93, 127.10, 132.27, 134.06, 145.41, 147.76, 150.22, 150.31, 158.65. MS (ESI positive) *m/z* = 505.3 [M + H]^+^. Elemental analysis calculated for C_21_H_20_N_4_O_9_S, C, 50.00; H, 4.00; N, 11.11; O, 28.54; S, 6.36; found C, 50.17, H, 3.98, N, 11.18, O, 28.58, S, 6.00.

#### 3.1.2. Preparation of ethyl 5-(4-(2-(2,4-dinitrophenylsulfonamido)acetyl)piperazin-1-yl)benzofuran-2-carboxylate (4b)

Using 500 mg of ethyl 5-(4-(2-aminoacetyl)piperazin-1-yl)benzofuran-2-carboxylate **3a** to get 330 mg, yield 39%, brown solid. ^1^H NMR (400 MHz, DMSO-d_6_) *δ* 1.32 (t, 3H, CH_3_), 3.04 (br s, 2H, piperazine-CH_2_), 3.13 (br s, 2H, piperazine-CH_2_), 3.53 (br s, 4H, piperazine-2CH_2_), 4.09 (s, 2H, acetyl-CH_2_), 4.31–4.3 (d, 2H, O-CH_2_), 7.19 (s, 1H, Ar-H), 7.27–7.30 (d, 1H, *J* = 9.00 Hz, Ar-H), 7.58–7.61 (d, 4H, *J* = 9.60 Hz, Ar-H), 7.63 (s, 1H, NH), 8.29–8.32 (d, 1H, *J* = 8.70, Ar-H), 8.57 (s, Ar-H), 8.63–8.66 (d, 1H, *J* = 8.70, Ar-H), 8.87 (s, 1H, Ar-H); ^13^C NMR (400 MHz, DMSO-d_6_) 14.13, 41.46, 44.02, 44.29, 49.60, 49.91, 59.75, 61.10, 108.20, 112.37, 114.13, 119.88, 120.06, 127.15, 131.82, 138.56, 145.37, 147.31, 148.20, 149.40, 150.19, 158.70, 165.70. MS (ESI positive) *m/z* = 562.2 [M + H]^+^. Elemental analysis calculated for C_23_H_23_N_5_O_10_S, C, 49.20; H, 4.13; N, 12.47; O, 28.49; S, 5.71; found C, 49.35, H, 4.20, N, 12.49, O, 28.48, S, 5.48.

#### 3.1.3. Preparation of ethyl 5-(4-(3-(2,4-dinitrophenyl sulfonamido)propanoyl)piperazin-1-yl) benzofuran-2-carboxylate (4c)

Using 500 mg of ethyl 5-(4-(3-aminopropanoyl)piperazin-1-yl)benzofuran-2-carboxylate **3b** to get 220 mg, yield 26%, brown syrup. ^1^H NMR (300 MHz, DMSO-d_6_) *δ* 1.31–1.35 (t, 3H, *J* = 6.90 Hz, CH_3_), 2.59–2.64 (t, 2H, *J* = 6.3 Hz, COCH_2_), 3.06 (br s, 2H, piperazine-CH_2_), 3.12 (br s, 2H, piperazine-CH_2_), 3.18–3.22 (2H, *J* = 6.0 Hz, N-CH_2_), 3.57 (br s, 4H, 2 piperizine-CH_2_), 4.31–4.38 (q, 2H, *J* = 6.9 Hz, CH_2_), 7.19 (s, 1H, Ar-H), 7.28–7.31 (d, 1H, *J* = 9.3 Hz, Ar-H), 7.59–7.62 (d, 1H, *J* = 9.3 Hz, Ar-H), 7.63 (br s, 1H, NH), 8.27–8.30 (d, 1H, *J* = 8.4 Hz, Ar-H), 8.46 (s, 1H, Ar-H), 8.65–8.67 (d, 1H, *J* = 8.7 Hz, Ar-H), 8.91 (s, 1H, Ar-H); ^13^C NMR (300 MHz, DMSO-d_6_) 14.13, 32.53, 38.23, 40.97, 44.68, 49.67, 50.06, 61.09, 108.16, 112.37, 114.14, 120.06, 120.10, 127.15, 127.30, 131.34, 137.68, 145.35, 147.69, 148.27, 149.64, 150.18, 158.70, 161.86, 168.30. MS (ESI positive) *m/z* = 576.2 [M + H]^+^. Elemental analysis calculated for C_24_H_25_N_5_O_10_S, C, 50.08; H, 4.38; N, 12.17; O, 27.80; S, 5.57; found C, 50.07, H, 4.38, N, 12.18, O, 27.79, S, 5.58.

#### 3.1.4. Preparation of ethyl 5-(4-(4-(2,4-dinitrophenyl sulfonamido)butanoyl)piperazin-1-yl) benzofuran-2-carboxylate (4d)

Using 500 mg of ethyl 5-(4-(4-aminobutanoyl)piperazin-1-yl)benzofuran-2-carboxylate **3c** to get 240 mg, yield 29% as yellow solid. ^1^H NMR (400 MHz, DMSO-d_6_) *δ* 1.32 (t, 3H, *J* = 7.2 Hz, CH_3_), 1.69 (q, 2H, *J* = 7.2 Hz, CH_2_), 2.37 (t, 2H, *J* = 7.2 Hz, CH_2_), 2.99 (t, 2H, *J* = 7.2 Hz, CH_2_), 3.05 (br s, 2H, piperazine-CH_2_), 3.06 (br s, 2H, piperazine-CH_2_), 3.55 (br s, 2H, piperazine-CH_2_), 3.59 (br s, 2H, piperazine-CH_2_), 4.35 (q, 2H, *J* = 7.2 Hz, CH_2_), 7.20 (d, 1H, *J* = 2.4 Hz, Ar-H), 7.28 (dd, 1H, *J* = 9.3, 2.8 Hz, Ar-H), 7.59 (d, 1H, *J* = 9.2 Hz, Ar-H), 7.62 (s, 1H, Ar-H), 8.24 (d, 1H, *J* = 8.4 Hz, Ar-H), 8.49 (t, 1H, *J* = 5.6 Hz, NH), 8.65 (dd, 1H, *J* = 8.8, 2.0 Hz, Ar-H), 8.89 (d, 1H, *J* = 2.4 Hz, Ar-H); ^13^C NMR (400 MHz, DMSO-d_6_): 14.10, 24.78, 29.02, 42.39, 50.09, 61.05, 108.14, 112.32, 114.10, 120.02, 127.12, 127.24, 131.23, 137.70, 145.32, 147.60, 148.27, 149.61, 150.15, 158.67, 169.84. MS (ESI positive) *m/z* = 590.1 [M + H]^+^. Elemental analysis calculated for C_25_H_27_N_5_O_10_S, C, 50.08; H, 4.38; N, 12.17; O, 27.80; S, 5.57; found C, 50.08, H, 4.38, N, 12.19, O, 27.80, S, 5.56.

#### 3.1.5. Preparation of ethyl 5-(4-(1-((2,4-dinitrophenyl)sulphonyl) piperidine-4-carbonyl)piperazin-1-yl)benzofuran-2-carboxylate (4e)

Using 500 mg of ethyl 5-(4-(piperidine-4-carbonyl) piperazin-1-yl)benzofuran-2-carboxylate **3d** to get 340 mg, yield 42% as yellow solid. ^1^H NMR (400 MHz, DMSO-d_6_) *δ* 1.32 (t, 3H, *J* = 7.2 Hz, CH_3_), 1.56 (d, 2H, *J* = 11.6 Hz, CH_2_), 1.77 (d, 2H, *J* = 11.6 Hz, CH_2_), 2.84–2.96 (m, 3H, piperazine-CH_2_ and piperazine CH), 3.06 (br s, 2H, piperazine-CH_2_), 3.11 (br s, 2H, piperazine-CH_2_), 3.61 (br s, 2H, piperazine-CH_2_), 3.65 (br s, 2H, piperazine-CH_2_), 3.74 (d, 2H, *J* = 12.4 Hz, piperazine-CH_2_), 4.34 (q, 2H, *J* = 7.2 Hz, CH_2_), 7.20 (d, 1H, *J* = 2.4 Hz, Ar-H), 7.29 (dd, 1H, *J* = 9.3, 2.8 Hz, Ar-H), 7.59 (d, 1H, *J* = 9.2 Hz, Ar-H), 7.62 (s, 1H, Ar-H), 8.30 (d, 1H, *J* = 8.4 Hz, Ar-H), 8.60 (dd, 1H, *J* = 8.8, 2.0 Hz, Ar-H), 9.01 (d, 1H, *J* = 2.4 Hz, Ar-H); ^13^C NMR (400 MHz, DMSO-d_6_): 14.09, 27.84, 35.62, 45.04, 50.43, 61.05, 108.22, 112.32, 114.09, 119.92, 120.05, 126.78, 127.11, 132.22, 134.58, 145.33, 147.68, 148.24, 150.06, 150.16, 158.66, 171.76. MS (ESI positive) *m/z* = 616.2 [M + H]^+^. Elemental analysis calculated for C_27_H_29_N_5_O_10_S, C, 52.68; H, 4.75; N, 11.38; O, 25.99; S, 5.21; found C, 52.68, H, 4.75, N, 11.39, O, 25.98, S, 5.20.

#### 3.1.6. Preparation of ethyl 5-(4-(1-((2,4-dinitrophenyl)sulphonyl)piperidine-3-carbonyl)piperazin-1-yl)benzofuran-2-carboxylate (4f)

Using 500 mg of ethyl 5-(4-(piperidine-3-carbonyl) piperazin-1-yl)benzofuran-2-carboxylate **3e** to get 220 mg, yield 27% as off brown solid. ^1^H NMR (DMSO-d_6_) *δ* 1.33 (t, 3H, *J* = 7.2 Hz, CH_3_), 1.62 (m, 2H, piperidine-CH_2_), 1.80 (m, 2H, piperidine-CH_2_), 2.94 (m, 1H, piperidine-CH), 3.10 (br-d, 4H, piperazine-2x CH_2_), 3.62–3.76 (m, 8H, piperazine-2x CH_2_, piperidine-2x CH_2_), 3.35 (q, 2H, *J* = 6.8 Hz, CH_2_), 7.22 (d, H, *J* = 2.4 Hz, Ar-H), 7.30 (dd, 1H, *J* = 9.20 and 2.0 Hz, Ar-H), 7.59–7.63 (m, 2H, 2 _ Ar-H), 8.29 (d, 1H, *J* = 8.8 Hz, Ar-H), 8.58 (dd, 1H, *J* = 8.8 and 2.4 Hz, Ar-H), 8.99 (d, 1H, *J* = 2.4 Hz, Ar-H); ^13^C NMR (400 MHz, DMSO-d_6_): 14.58, 24.45, 27.17, 38.10, 41.50, 45.23, 46.43, 48.45, 50.24, 50.88, 61.54, 108.73, 112.82, 114.59, 120.43, 120.55, 127.39, 127.61, 132.48, 135.26, 145.82, 148.15, 148.71, 150.55, 150.66, 159.16, 170.87. MS (ESI positive) *m/z* = 616.2 [M + H]^+^. Elemental analysis calculated for C_27_H_29_N_5_O_10_S, C, 52.68, H, 4.75, N, 11.38, O, 25.99, S, 5.21, found C, 52.67, H, 4.76, N, 11.38, O, 25.99, S, 5.21.

#### 3.1.7. Preparation of (S)-ethyl 5-(4-(1-((2,4-dinitrophenyl)sulphonyl)pyrrolidine-2-carbonyl) piperazin-1-yl)benzofuran-2-carboxylate (4g)

Using 500 mg of (S)-ethyl 5-(4-(pyrrolidine-2-carbonyl)piperazin-1-yl)benzofuran-2-carboxylate **3f** to get 190 mg, yield 31% as off brown solid. ^1^H NMR (400 MHz, DMSO-d_6_) *δ* 1.33 (t, 3H, *J* = 7.2 Hz, CH_3_), 1.86–1.94 (m, 3H, pyrrolidine-CH_2_ and 0.5 pyrrolidine-CH_2_), 2.28–2.30 (m, 1H, 0.5 pyrrolidine-CH_2_), 3.06, 3.17 (m, 4H, 2x piperazine-CH_2_), 3.51–3.75 (m, 4H, 2x piperazine-CH_2_ and pyrrolidine-CH_2_), 4.35 (q, 2H, *J* = 7.2 Hz, CH_2_), 5.04 (m, 1H, pyrrolidine-CH), 7.23 (d, 1H, *J* = 2.4 Hz, Ar-H), 7.31 (dd, 1H, *J* = 9.3, 2.8 Hz, Ar-H), 7.61 (d, 1H, *J* = 9.2 Hz, Ar-H), 7.63 (s, 1H, Ar-H), 8.38 (d, 1H, *J* = 8.4 Hz, Ar-H), 8.58 (dd, 1H, *J* = 8.8, 2.0 Hz, Ar-H), 8.91 (d, 1H, *J* = 2.4 Hz, Ar-H); ^13^C NMR (400 MHz, DMSO-d_6_): 14.09, 24.03, 30.55, 41.58, 44.66, 48.74, 49.70, 50.04, 59.16, 61.06, 108.28, 112.34, 114.10, 119.68, 120.09, 126.66, 127.13, 131.96, 136.47, 145.35, 147.61, 148.20, 149.64, 150.20, 158.67, 168.55. MS (ESI positive) *m/z* = 602.1 [M + H]^+^. Elemental analysis calculated for C_26_H_27_N_5_O_10_S, C, 51.91; H, 4.52; N, 11.64; O, 26.60; S, 5.33; S, 5.21; found C, 51.90, H, 4.53, N, 11.65, O, 26.60, S, 5.22.

#### 3.1.8. Preparation of (R)-ethyl 5-(4-(1-((2,4-dinitrophenyl)sulphonyl)pyrrolidine-2-carbonyl) piperazin-1-yl)benzofuran-2-carboxylate (4h)

Using 500 mg of (R)-ethyl 5-(4-(pyrrolidine-2-carbonyl)piperazin-1-yl) benzofuran-2-carboxylate **3g** to get 185 mg, yield 30% as yellow solid. ^1^H NMR (400 MHz, DMSO-d_6_) *δ* 1.34 (t, 3H, *J* = 7.2 Hz, CH_3_), 1.85–2.04 (m, 4H, 2x pyrrolidine-CH_2_), 3.07, 3.17 (m, 4H, 2x piperazine-CH_2_), 3.40–3.72 (m, 4H, 2x piperazine-CH_2_ and pyrrolidine-CH_2_), 4.35 (q, 2H, *J* = 7.2 Hz, CH_2_), 5.09 (m, 1H, pyrrolidine-CH), 6.98 (d, 1H, *J* = 8.4 Hz, Ar-H), 7.25 (d, 1H, *J* = 2.4 Hz, Ar-H), 7.33 (dd, 1H, *J* = 9.3, 2.8 Hz, Ar-H), 7.62 (d, 1H, *J* = 9.2 Hz, Ar-H), 7.64 (s, 1H, Ar-H), 8.23 (dd, 1H, *J* = 8.8, 2.0 Hz, Ar-H), 8.52 (d, 1H, *J* = 2.4 Hz, Ar-H); MS (ESI positive) *m/z* = 602.1 [M + H]^+^. Elemental analysis calculated for C_26_H_27_N_5_O_10_S, C, 51.91; H, 4.52; N, 11.64; O, 26.60; S, 5.33; found C, 51.91, H, 4.51, N, 11.65, O, 26.60, S, 5.33.

#### 3.1.9. Preparation of ethyl 5-(4-(1-((2,4-dinitrophenyl)sulphonyl)pyrrolidine-3-carbonyl) piperazin-1-yl)benzofuran-2-carboxylate (4i)

Using 500 mg of ethyl 5-(4-(pyrrolidine-3-carbonyl)piperazin-1-yl)benzofuran-2-carboxylate **3f** to get 220 mg, yield 36% as yellow solid. ^1^H NMR (400 MHz, DMSO-d_6_) *δ* 1.33 (t, 3H, *J* = 6.8 Hz, CH_3_), 1.97 (m, 1H, 0.5x pyrrolidine-CH_2_), 2.16 (m, 1H, 0.5x pyrrolidine-CH_2_), 3.07 (br s, 2H, piperazine-CH_2_), 3.13 (br s, 2H, piperazine-CH_2_), 3.43–3.66 (m, 9H, piperazine-CH_2,_ 2x pyrrolidine-CH_2_, pyrrolidine-CH), 4.34 (q, 2H, *J* = 6.8 Hz, CH_2_), 7.21 (d, 1H, *J* = 2.4 Hz, Ar-H), 7.29 (dd, 1H, *J* = 9.3, 2.8 Hz, Ar-H), 7.60 (d, 1H, *J* = 9.2 Hz, Ar-H), 7.62 (s, 1H, Ar-H), 8.33 (d, 1H, *J* = 8.4 Hz, Ar-H), 8.58 (dd, 1H, *J* = 8.8, 2.0 Hz, Ar-H), 8.99 (d, 1H, *J* = 2.4 Hz, Ar-H); ^13^C NMR (400 MHz, DMSO-d_6_): 14.15, 28.92, 41.36, 44.83, 47.83, 49.73, 50.20, 61.12, 108.26, 112.39, 114.17, 119.92, 120.14, 132.00, 134.58, 145.36, 147.87, 148.25, 149.96, 150.21, 158.72, 169.63. MS (ESI positive) *m/z* = 602.1 [M + H]^+^. Elemental analysis calculated for C_26_H_27_N_5_O_10_S, C, 51.91; H, 4.52; N, 11.64; O, 26.60; S, 5.33; found C, 51.91, H, 4.52, N, 11.64, O, 26.60, S, 5.33.

#### 3.1.10. Preparation of ethyl 5-(4-(2-(2,4-dinitrophenyl sulfonamido)-2-methylpropanoyl) piperazin-1-yl)benzofuran-2-carboxylate (4j)

Using 500 mg of ethyl 5-(4-(2-amino-2-methyl propanoyl)piperazin-1-yl)benzofuran-2-carboxylate **3i** to get 210 mg, yield 25% as yellow solid. ^1^H NMR (400 MHz, DMSO-d_6_) *δ* 1.33 (t, 3H, *J* = 7.2 Hz, CH_3_), 3.24 (br s, 4H, 2x 2x piperazine-CH_2_), 3.31 (s, 6H, 2x CH_3_), 3.42 (br s, 4H, 2x piperazine-CH_2_), 4.34 (q, 2H, *J* = 6.8 Hz, CH_2_), 7.21 (d, 1H, *J* = 2.4 Hz, Ar-H), 7.27 (dd, 1H, *J* = 9.3, 2.8 Hz, Ar-H), 7.59 (d, 1H, *J* = 9.2 Hz, Ar-H), 7.61 (s, 1H, Ar-H), 8.32 (d, 1H, *J* = 8.4 Hz, Ar-H), 8.59 (dd, 1H, *J* = 8.8, 2.0 Hz, Ar-H), 9.01 (d, 1H, *J* = 2.4 Hz, Ar-H); ^13^C NMR (400 MHz, DMSO-d_6_): 14.09, 45.72, 49.61, 61.08, 108.64, 112.40, 114.06, 120.02, 120.29, 126.92, 127.09, 132.26, 134.08, 145.40, 147.75, 150.21, 150.31, 158.63. MS (ESI positive) *m/z* = 590.2 [M + H]^+^. Elemental analysis calculated for C_25_H_27_N_5_O_10_S, C, 50.93; H, 4.62; N, 11.88; O, 27.14; S, 5.44; found C, 50.93, H, 4.60, N, 11.90, O, 27.15, S, 5.43.

#### 3.1.11. Preparation of ethyl 5-(4-(2-(2,4-dinitrophenyl sulfonamido)-2-phenylacetyl)piperazin-1-yl) benzofuran-2-carboxylate (4k)

Using 500 mg of ethyl 5-(4-(2-amino-2-phenylacetyl) piperazin-1-yl)benzofuran-2-carboxylate **3j** to get 230 mg, yield 29% as brown solid. ^1^H NMR (400 MHz, DMSO-d_6_) *δ* 1.33 (t, 3H, *J* = 6.8 Hz, CH_3_), 2.98 (br s, 4H, 2x piperazine-CH_2_), 3.56 (br s, 2H, piperazine-CH_2_), 3.63 (br s, 2H, piperazine-CH_2_), 4.33 (q, 2H, *J* = 6.8 Hz, CH_2_), 5.60 (d, 1H, *J* = 8.4 Hz, CH), 7.08 (d, 1H, *J* = 2.0 Hz, Ar-H), 7.14–7.33 (m, 7H, 7x Ar-H), 7.55 (d, 1H, *J* = 9.2 Hz, Ar-H), 7.59 (s, 1H, Ar-H), 8.09 (d, 1H, *J* = 9.2 Hz, Ar-H), 8.45 (dd, 1H, *J* = 8.8, 2.0 Hz, Ar-H), 8.80 (d, 1H, *J* = 2.0 Hz, Ar-H), 9.13 (d, 1H, *J* = 8.4 Hz, NH); MS (ESI positive) *m/z* = 638.5 [M + H]^+^. Elemental analysis calculated for C_29_H_27_N_5_O_10_S, C, 54.63; H, 4.27; N, 10.98; O, 25.09; S, 5.03; found C, 54.63, H, 4.27, N, 10.99, O, 25.08, S, 5.05.

#### 3.1.12. Preparation of 5-(4-((2,4-dinitrophenyl)sulphonyl) piperazin-1-yl)benzofuran-2-carboxamide (4m)

Using 500 mg of 5-(piperazin-1-yl)benzofuran-2-carboxamide **7a** to get 240 mg, yield 25% as pale yellow solid. ^1^H NMR (400 MHz, DMSO-d_6_) *δ* 3.23 (br s, 4H, 2x piperazine-CH_2_), 3.41 (br z, 4H, 2x Piperazine-CH_2_), 7.16–7.21 (m, 2H, 2x Ar-H), 7.41 (s, 1H, Ar-H), 7.49 (d, 1H, *J* = 8.8 Hz, Ar-H), 7.60 (br s, 1H, 0.5x NH2), 8.02 (br s, 1H, 0.5x NH2), 8.33 (d, 1H, *J* = 8.8 Hz, Ar-H), 8.60 (dd, 1H, *J* = 8.8, 2.4 Hz, Ar-H), 9.01 (d, 1H, *J* = 2.4 Hz, Ar-H); ^13^C NMR (400 MHz, DMSO-d_6_): 45.75, 49.74, 108.73, 109.56, 111.98, 118.84, 120.02, 126.93, 127.66, 132.26, 134.08, 147.48, 147.81, 149.50, 149.69, 150.22, 159.76. MS (ESI positive) *m/z* = 477.5 [M + H]^+^. Elemental analysis calculated for C_19_H_17_N_5_O_8_S, C, 48.00; H, 3.60; N, 14.73; O, 26.92; S, 6.74; found C, 48.01, H, 3.61, N, 14.71, O, 26.92, S, 6.74.

#### 3.1.13. Preparation of 5-(4-((2,4-dinitrophenyl)sulphonyl) piperazin-1-yl)-N-phenylbenzofuran-2-carboxamide (4n)

Using 500 mg of N-phenyl-5-(piperazin-1-yl)benzofuran-2-carboxamide **7b** to get 250 mg, yield 29% as white solid. ^1^H NMR (400 MHz, DMSO-d_6_) *δ* 3.26 (br s, 4H, 2x piperazine-CH_2_), 3.43 (br s, 4H, 2x piperazine-CH_2_), 7.13 (t, 1H, *J* = 7.2 Hz, Ar-H), 7.21–7.26 (m, 2H, 2x Ar-H), 7.36 (t, 2H, *J* = 7.6 Hz, 2x Ar-H), 7.58 (d, 1H, *J* = 9.2 Hz, Ar-H), 7.64 (s, 1H, Ar-H), 7.80 (d, 1H, *J* = 8.0 Hz, Ar-H), 8.33 (d, 1H, *J* = 8.8 Hz, Ar-H), 8.60 (dd, 1H, *J* = 8.8, 2.4 Hz, Ar-H), 9.01 (d, 1H, *J* = 2.4 Hz, Ar-H), 10.43 (s, 1H, NH); ^13^C NMR (400 MHz, DMSO-d_6_): 45.76, 49.69, 108.76, 110.66, 112.14, 119.25, 120.46, 123.98, 126.93, 127.62, 128.63, 132.27, 134.08, 138.31, 147.66, 147.82, 149.14, 149.68, 150.22, 156.61. MS (ESI positive) *m/z* = 552.5 [M + H]^+^. Elemental analysis calculated for C_25_H_21_N_5_O_8_S C, 54.44; H, 3.84; N, 12.70; O, 23.21; S, 5.81; found C, 54.45, H, 3.84, N, 12.70, O, 23.21, S, 5.80.

#### 3.1.14. Preparation of (5-(4-((2,4-dinitrophenyl)sulphonyl) piperazin-1-yl)benzofuran-2-yl) (piperidin-1-yl)methanone (4o)

Using 500 mg of (5-(piperazin-1-yl)benzofuran-2-yl)(piperidin-1-yl)methanone **7c** to get 210 mg, yield 24% as white solid. ^1^H NMR (400 MHz, DMSO-d_6_) *δ* 1.56 (br s, 4H, 2x piperidine-CH_2_), 1.64 (br s, 2H, piperidine-CH_2_), 3.22 (br s, 4H, 2x piperazine-CH_2_), 3.41 (br s, 4H, 2x piperazine-CH_2_), 3.61 (br s, 4H, 2x piperidine-CH_2_), 7.14–7.20 (m, 3H, 3x Ar-H), 7.51 (d, 1H, *J* = 8.8 Hz, Ar-H), 8.32 (d, 1H, *J* = 8.8 Hz, Ar-H), 8.60 (dd, 1H, *J* = 8.8, 2.4 Hz, Ar-H), 9.01 (d, 1H, *J* = 2.4 Hz, Ar-H); ^13^C NMR (400 MHz, DMSO-d_6_): 23.97, 45.74, 49.80, 108.43, 110.12, 111.92, 118.38, 120.02, 126.92, 127.20, 132.26, 134.06, 147.54, 147.81, 149.03, 149.13, 150.21, 158.79. MS (ESI positive) *m/z* = 544.5 [M + H]^+^. Elemental analysis calculated for C_24_H_25_N_5_O_8_S C, 53.03; H, 4.64; N, 12.88; O, 23.55; S, 5.90; found C, 53.03, H, 4.65, N, 12.87, O, 23.55, S, 5.91.

#### 3.1.15. Preparation of (5-(4-((2,4-dinitrophenyl)sulphonyl) piperazin-1-yl)benzofuran-2-yl)(4-methylpiperazin-1-yl) methanone (4p)

Using 500 mg of (4-methylpiperazin-1-yl)(5-(piperazin-1-yl)benzofuran-2-yl)methanone **7d** to get 230 mg, yield 27% as white solid. ^1^H NMR (400 MHz, DMSO-d_6_) *δ* 2.22 (s, 3H, CH_3_), 2.38 (br s, 4H, 2x piperazine-CH_2_), 3.21 (br s, 4H, 2x piperazine-CH_2_), 3.42 (br s, 4H, 2x piperazine-CH_2_), 3.68 (br s, 4H, 2x piperidine-CH_2_), 7.15–7.18 (m, 3H, 3x Ar-H), 7.26 (s, 1H, Ar-H), 7.52 (d, 1H, *J* = 8.8 Hz, Ar-H), 8.32 (d, 1H, *J* = 8.8 Hz, Ar-H), 8.60 (dd, 1H, *J* = 8.8, 2.4 Hz, Ar-H), 9.01 (d, 1H, *J* = 2.4 Hz, Ar-H); ^13^C NMR (400 MHz, DMSO-d_6_): 45.39, 45.73, 49.78, 54.48, 108.44, 110.89, 111.98, 118.58, 120.01, 126.90, 127.13, 132.26, 134.07, 147.57, 147.81, 148.81, 149.12, 150.19. MS (ESI positive) *m/z* = 559.4′ [M + H]^+^. Elemental analysis calculated for C_24_H_26_N_6_O_8_S C, 51.61; H, 4.69; N, 15.05; O, 22.92; S, 5.74; found C, 51.60, H, 4.69, N, 15.06, O, 22.92, S, 5.74.

## Supplementary Material

Supplemental Material
