# Supplementary material for: Design and characterisation of piperazine-benzofuran integrated dinitrobenzenesulfonamide as Mycobacterium tuberculosis H37Rv strain inhibitors
Source: J Enzyme Inhib Med Chem. 2021 Jul 29;36(1):1751–9. doi: 10.1080/14756366.2021.1956914 (PMC8330757; doi:10.1080/14756366.2021.1956914)
Supplement: Supplemental Material [file IENZ_A_1956914_SM2656.pdf]

Supporting Information

Design and Characterization of Piperazine-benzofuran integrated Dinitrobenzenesulfonamide as *Mycobacterium tuberculosis* H37Rv strain inhibitors

Vallabhaneni S. Murthy[a], Yasinalli Tamboli[a], Vagolu Siva Krishna[b], Dharmarajan Sriram[b], Siddique Akber Ansari[c], Abdullah A. Alarfaj[d], Abdurahman H. Hirad[d], Vijayaparthasarathi Vijayakumar[a*]

[a] *Centre for Organic and Medicinal Chemistry, VIT University, Vellore - 632 014, Tamil Nadu, India.*
[b] *Medicinal Chemistry and Antimycobacterial Research Laboratory, Pharmacy Group, Birla Institute of Technology & Science Pilani, Hyderabad Campus, Hyderabad 500078, Telangana, India.*
[c] *Department of Pharmaceutical Chemistry, College of Pharmacy, King Saud University, Riyadh, P.O. Box 2454,Riyadh 11451, Saudi Arabia*
[d] *Department of Botany and Microbiology, College of Science, King Saud University, P.O. Box: 2455 Riyadh, 11451, Saudi Arabia*

Table of contents

1. Experimental section

2. Spectral data of synthesized compounds

3. References

1. Experimental Section

1.1 Chemistry

1.1. ***General procedure for the synthesis of 2a-2j:*** To solution of Ethyl 5-(piperazin-1-yl) benzofuran-2-carboxylate (1 eq) in DMF (10 vol), Amino acid (1.1 eq), HATU (1.5 eq) and DIPEA (3 eq) were added and stirred at 25°C for 2h. TLC showed the completion of starting material and formation of non-polar spot. The reaction mixture was quenched with water, extracted with ethyl acetate. The obtained crude product was purified by silica gel (100-200 mesh) column chromatography using ethyl acetate in hexane as eluent to give **2a-2j**

1.2. ***General procedure for the synthesis of 3a-3j:*** To solution of appropriate **2a-2j** derivatives (1 eq) in DCM (10 vol), TFA (4 eq) were added and stirred at 25°C for 2h. TLC showed the completion of starting material and formation of polar spot. The reaction mixture was concentrated dry to give **3a-3j,** the obtained crude was used directly in next step.

1.3. ***Preparation of 5-(4-(tert-butoxycarbonyl)piperazin-1-yl)benzofuran-2-carboxylic acid (*****5):** To solution of tert-butyl 4-(2-(ethoxycarbonyl)benzofuran-5-yl)piperazine-1-carboxylate **1b** (1 eq) in THF (7 vol), water (2 vol),ethanol (1vol) and LiOH (1.5eq) were added and stirred at 25°C for 16h. TLC showed the completion of starting material and formation of polar spot. The reaction mixture was washed with ethyl acetate (decant the organic layer) and the separated aqueous layer acidified with 2N HCl, extracted with ethyl acetate, dried over sodium sulphate and concentrated to give Compound-5.

1.4. ***General procedure for the synthesis of 6a-6d:*** To solution of acid derivative **5** (1 eq) in DMF (10 vol), Amine (1.5eq), HATU (1.5eq) and DIPEA (3 eq) were added and stirred at 25°C for 2h. TLC showed the completion of starting material and formation of non-polar spot. The reaction mixture was quenched with water, extracted with ethyl acetate, dried over sodium sulphate, concentrated to dry and purified by silica gel (100-200 mesh) column chromatography using ethyl acetate in hexane as eluent to give **6a-6d.**

1.5. ***General procedure for the synthesis of 7a-7d:*** To solution of appropriate **6a-6d** derivatives (1 eq) in DCM (10 vol), TFA (3 eq) was added and stirred at 25°C for 2h. TLC showed the

completion of starting material and formation of polar spot. The reaction mixture was concentrated to dry to give **7a-7d,** used directly in next step.

1.2. In-vitro *Mtb* MABA assay

Briefly, the inoculum was prepared from fresh LJ medium re-suspended in 7H9-S medium (7H9 broth, 0.1% casitone, 0.5% glycerol, supplemented oleic acid, albumin, dextrose, and catalase [OADC]), adjusted to an OD_{590} 1.0, and diluted 1:20; 100 µl was used as inoculum. Each drug stock solution was thawed and diluted in 7H9-S at four-fold the final highest concentration tested. Serial two-fold dilutions of each drug were prepared directly in a sterile 96-well microtiter plate using 100 µl 7H9-S. A growth control containing no antibiotic and a sterile control were also prepared on each plate. Sterile water was added to all perimeter wells to avoid evaporation during the incubation. The plate was covered, sealed in plastic bags and incubated at 37 ^{o}C in normal atmosphere. After 7 days incubation, 30 µl of Alamar blue solution was added to each well, and the plate was re-incubated overnight. A change in colour from blue (oxidised state) to pink (reduced) indicated the growth of bacteria, and the MIC was defined as the lowest concentration of drug that prevented this change in colour.

1.3. Cytotoxicity assay

Most active anti-TB compounds were further examined for toxicity in human cell lines A549 at the concentration of 50 mg/mL. After 72 h of exposure, viability was assessed on the basis of cellular conversion of MTT into a formazan product using the Promega Cell Titer 96 non-radioactive cell proliferation assay.

1.4. DPPH radical scavenging activity

The hydrogen atom or electron donation ability of some compounds were measured from the bleaching of the purple colored methanol solution of 1,1-diphenyl-1-picrylhydrazyl (DPPH). The spectrophotometric assay uses the stable radical DPPH as a reagent. 1 mL of various concentrations of the test compounds (5, 10, 25, 50 and 100 µg/mL) in methanol was added to 4 mL of 0.004% (w/v) methanol solution of DPPH. The reaction mixture was incubated at 37 °C. The scavenging activity on DPPH was determined by measuring the absorbance at 517 nm after 30 min. All tests were performed in triplicate and the mean values were entered. The percent of inhibition (I %) of free radical production from DPPH was calculated by the following equation % of scavenging = [(A control – A sample)/ (A sample × 100)]. Where, A control is the

absorbance of the control (DPPH radical without test sample) A sample is the absorbance of the test sample (DPPH radical with test sample). The control contains all reagents except the test samples.

1.5. Molecular docking

Molecular Docking analysis was performed to identify the possible mechanism of action of synthesized derivatives. Structure of the Mycobacterium tuberculosis InhA bound with ETH-NAD adduct (PDB ID 2H9I) was downloaded from www.rcsb.org. The protein structure was prepared for docking analysis via removal of water molecules and addition of the hydrogen atoms. Ligand structures was prepared via molecule builder module of the V life MDS 4.6 and optimized using V life MDS 4.6 engine. Grip based docking simulations were performed keeping the number of placement, a number of placements were 30 and rotation angle at 10°. The docking interactions were analyzed using a free version of discovery studio visualizer downloaded from https://discover.3ds.com/discovery-studio-visualizer-download .

2. Spectral data of synthesized compounds

The 1H, 13C NMR and LC-MS Spectra of Compound 4a

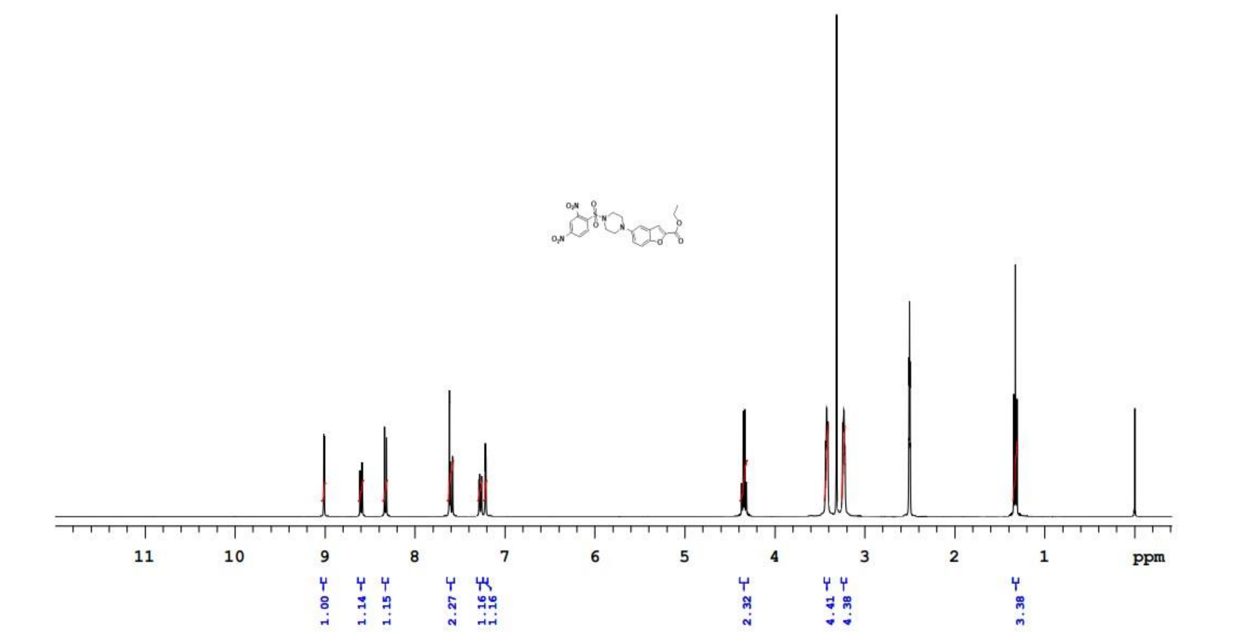

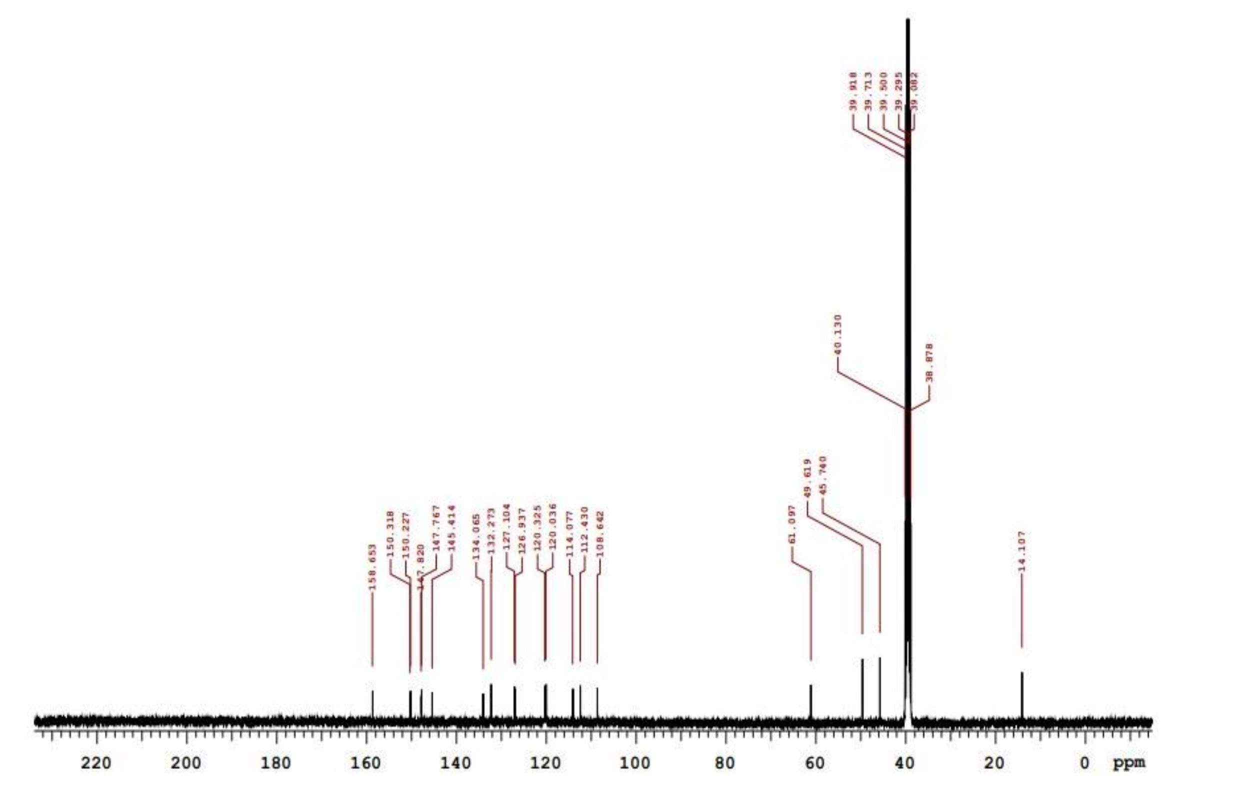

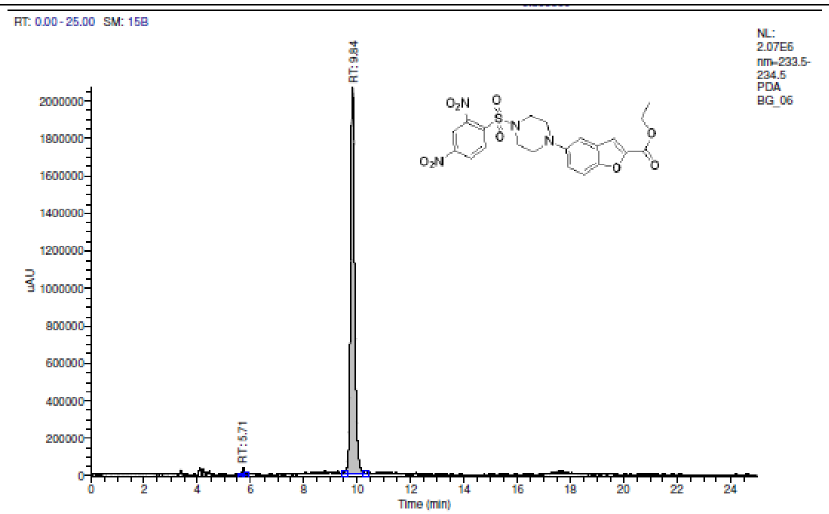

S.No.	Apex RT	Area	%Area
1	5.71	263384	1.22
2	9.84	21395148	98.78

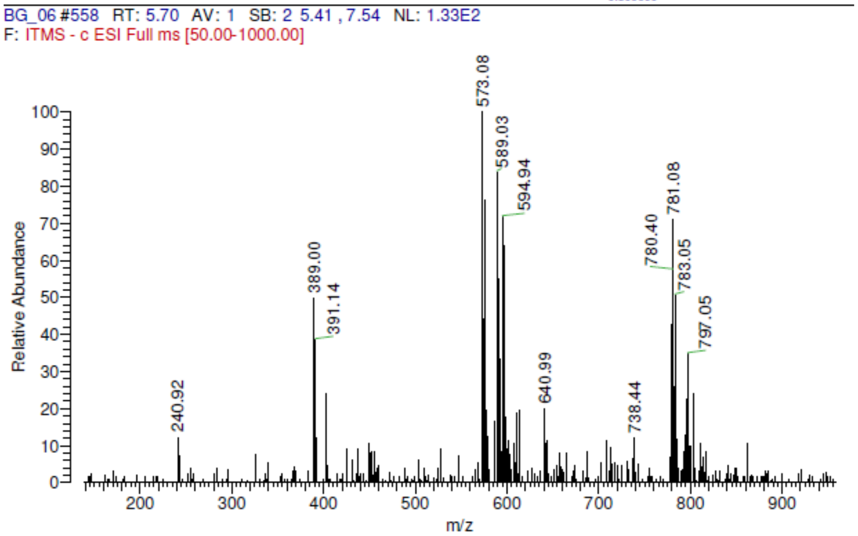

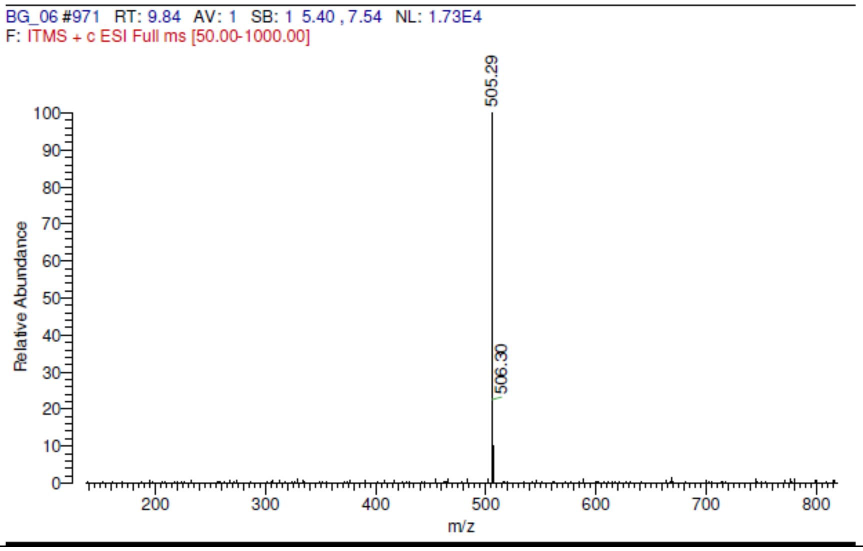

The 1H, 13C NMR Spectra of Compound 4b

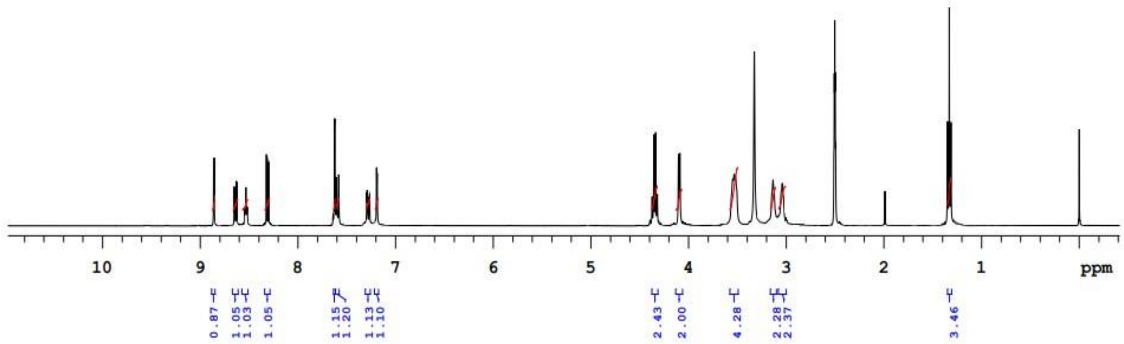

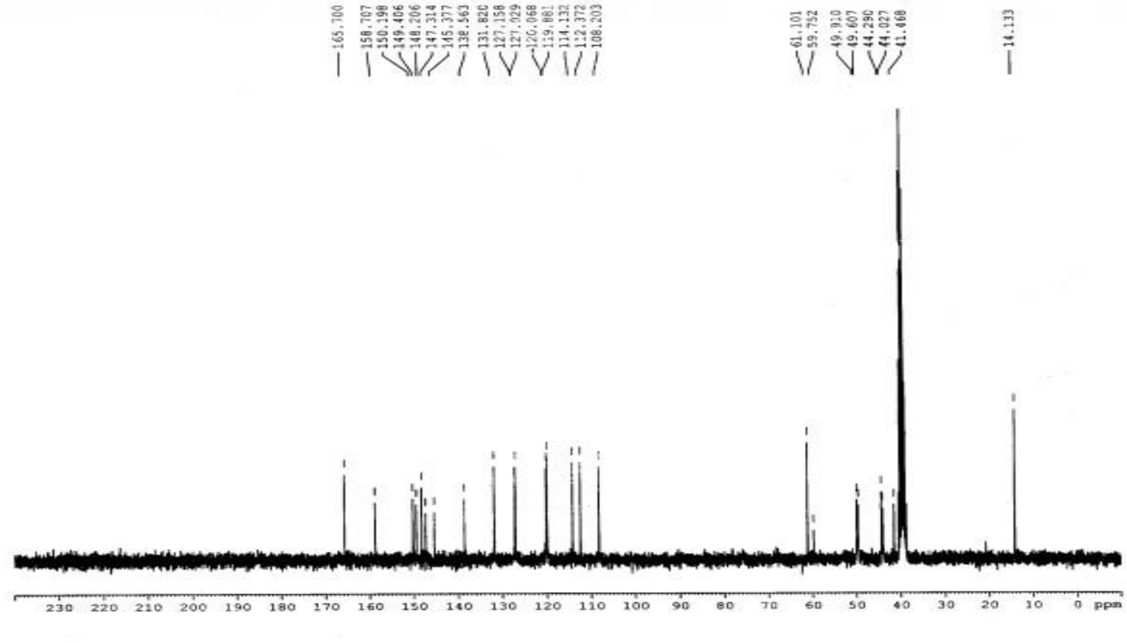

he 1H, 13C NMR of Compound 4c

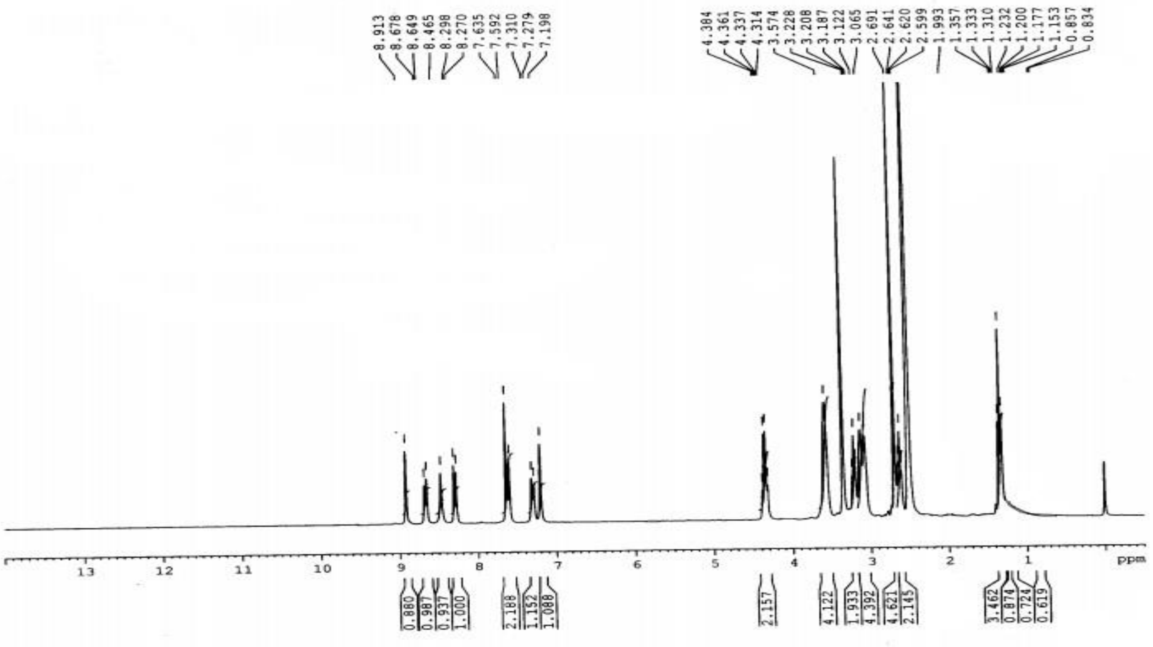

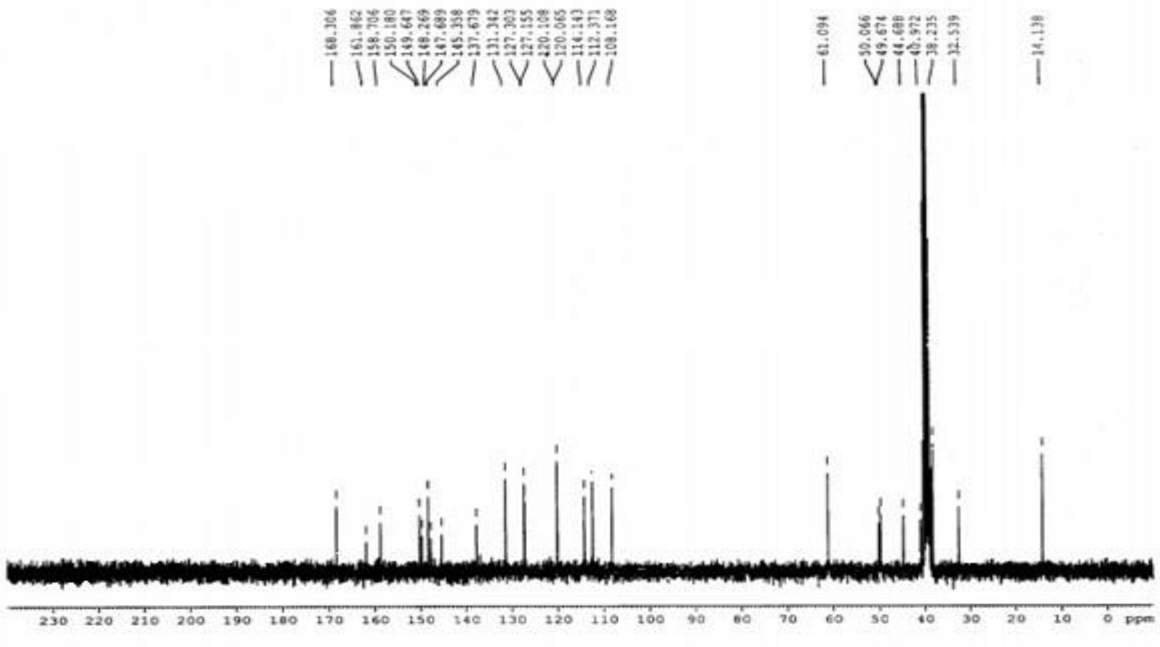

The 1H, 13C NMR of Compound 4d

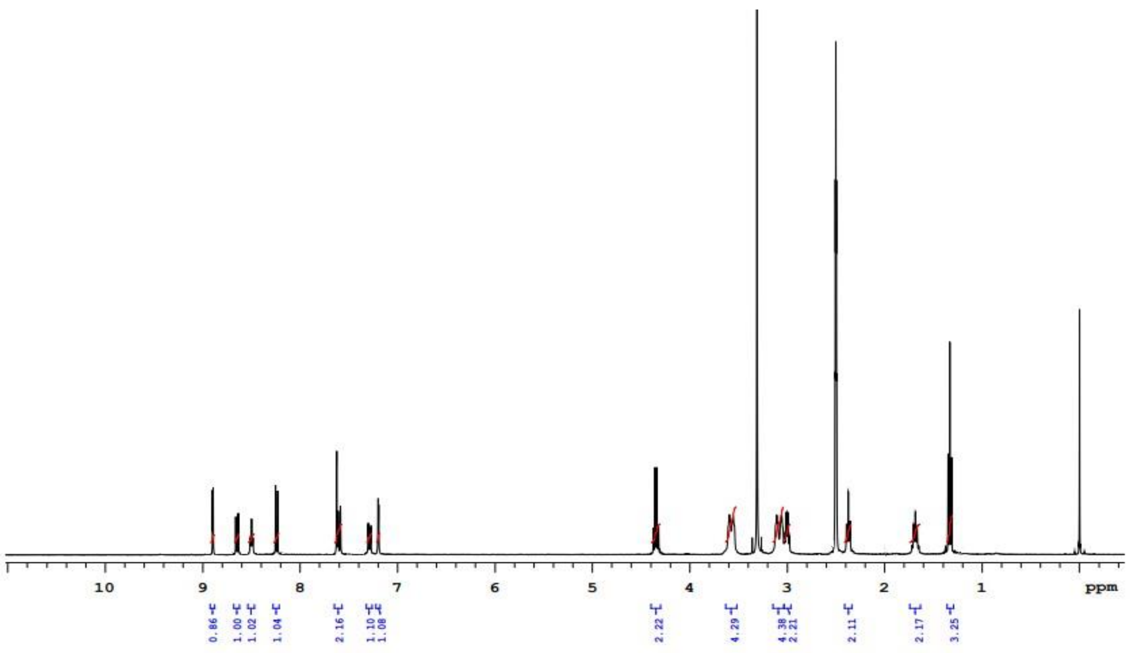

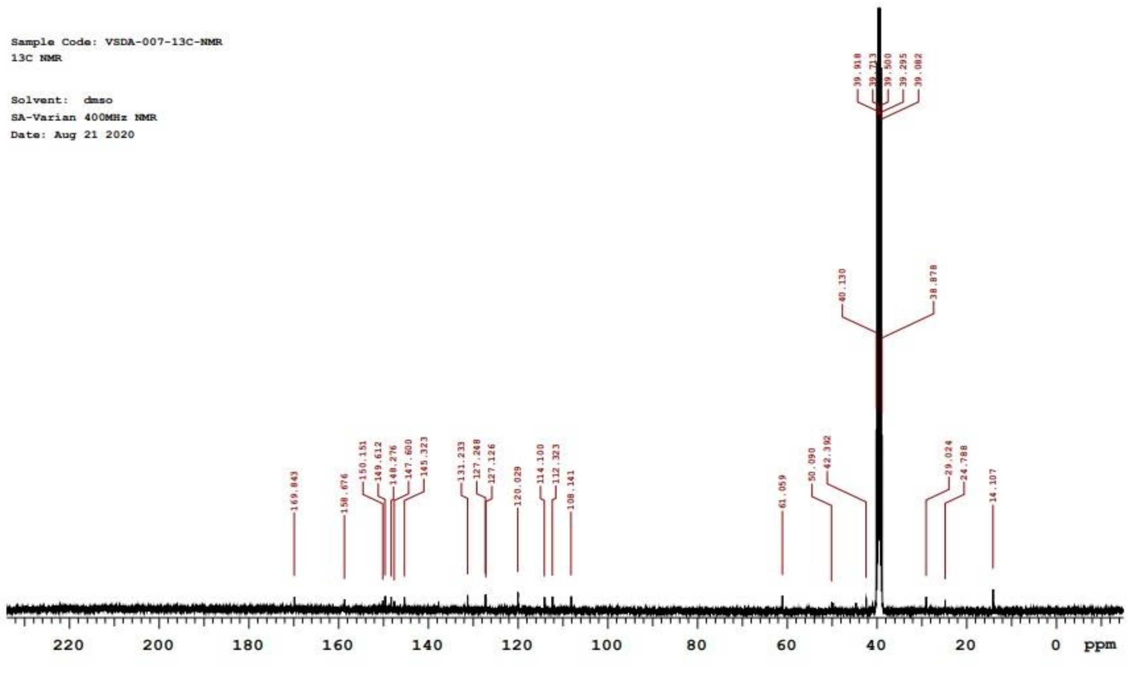

The 1H, 13C NMR of Compound 4e

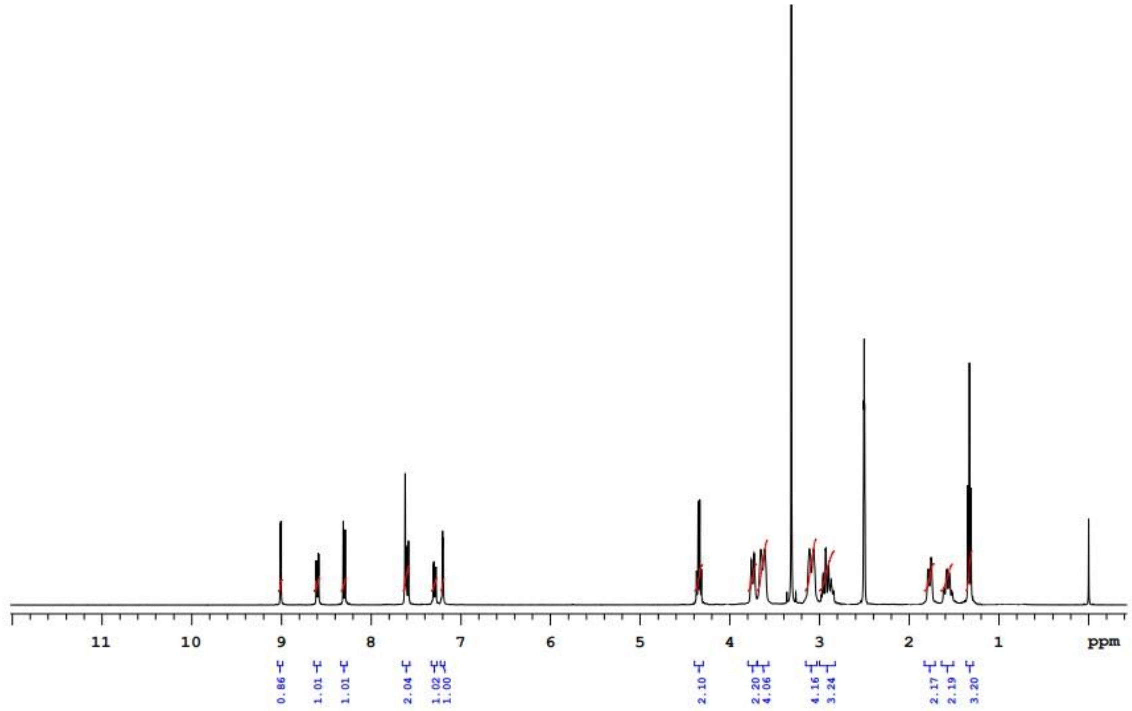

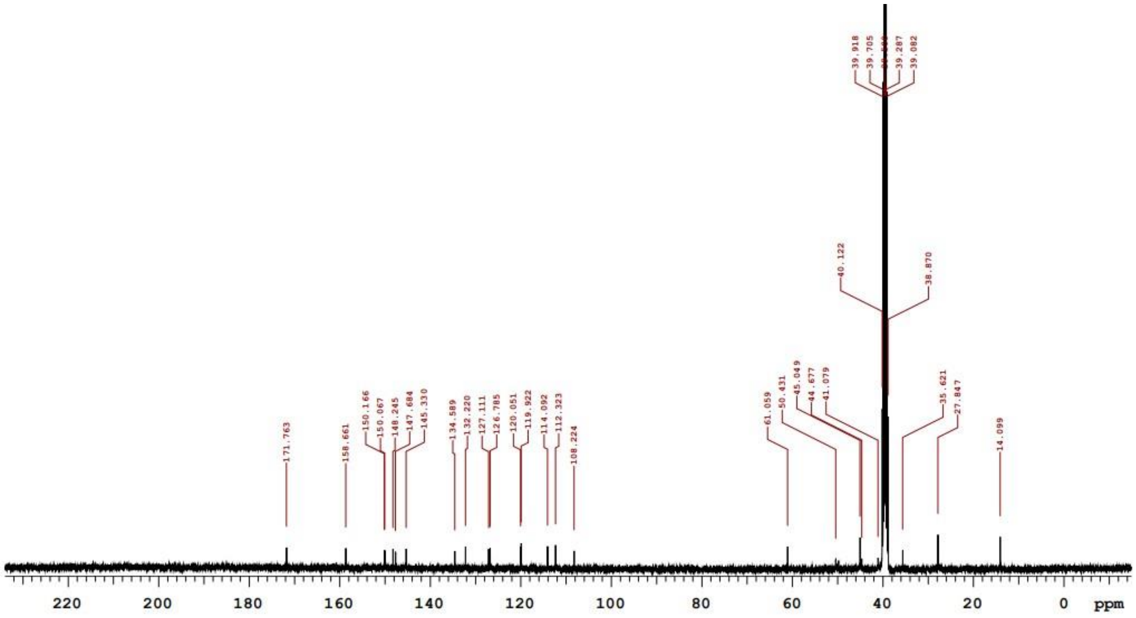

The 1H, 13C NMR of Compound 4f

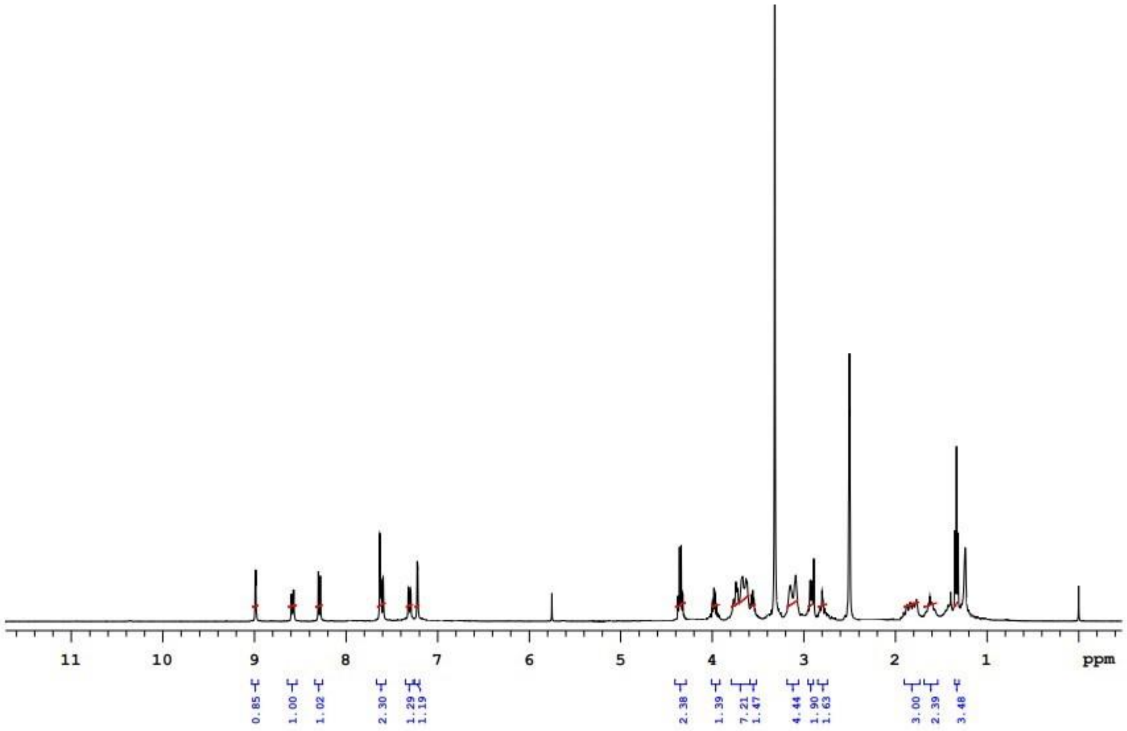

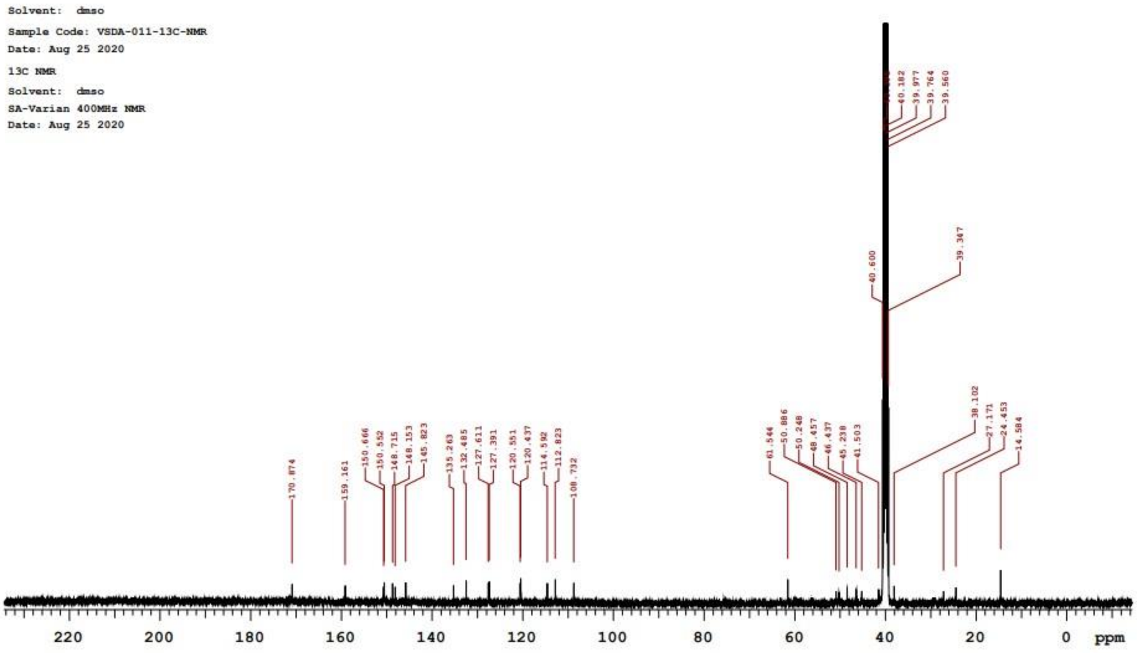

The 1H, 13C NMR of Compound 4g

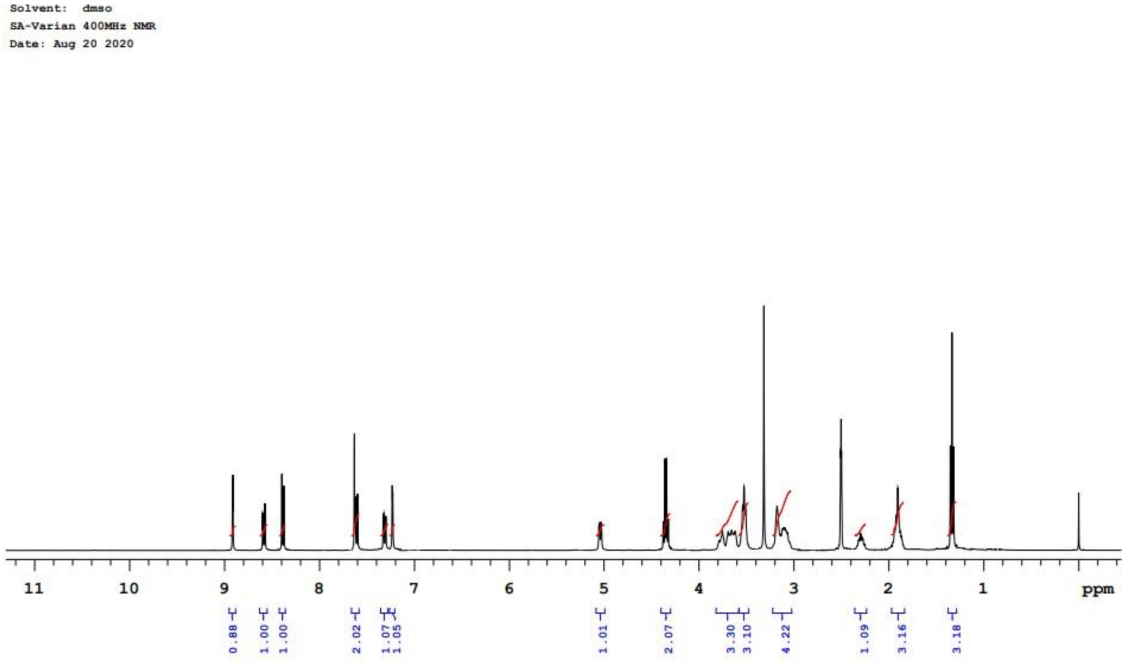

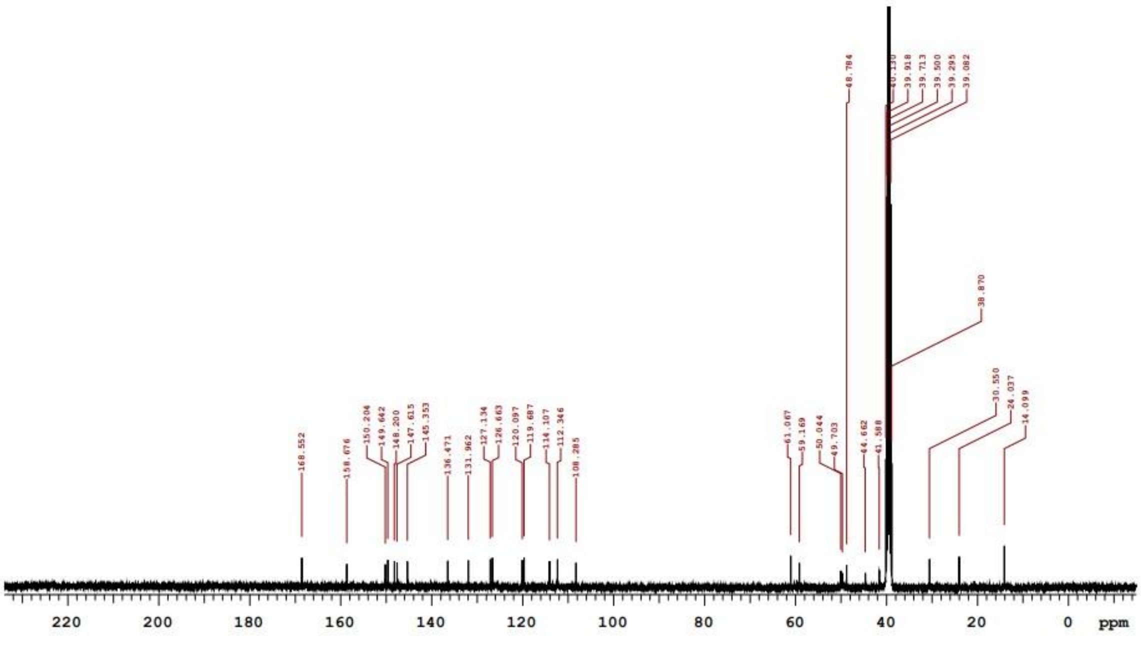

The 1H NMR of Compound 4h

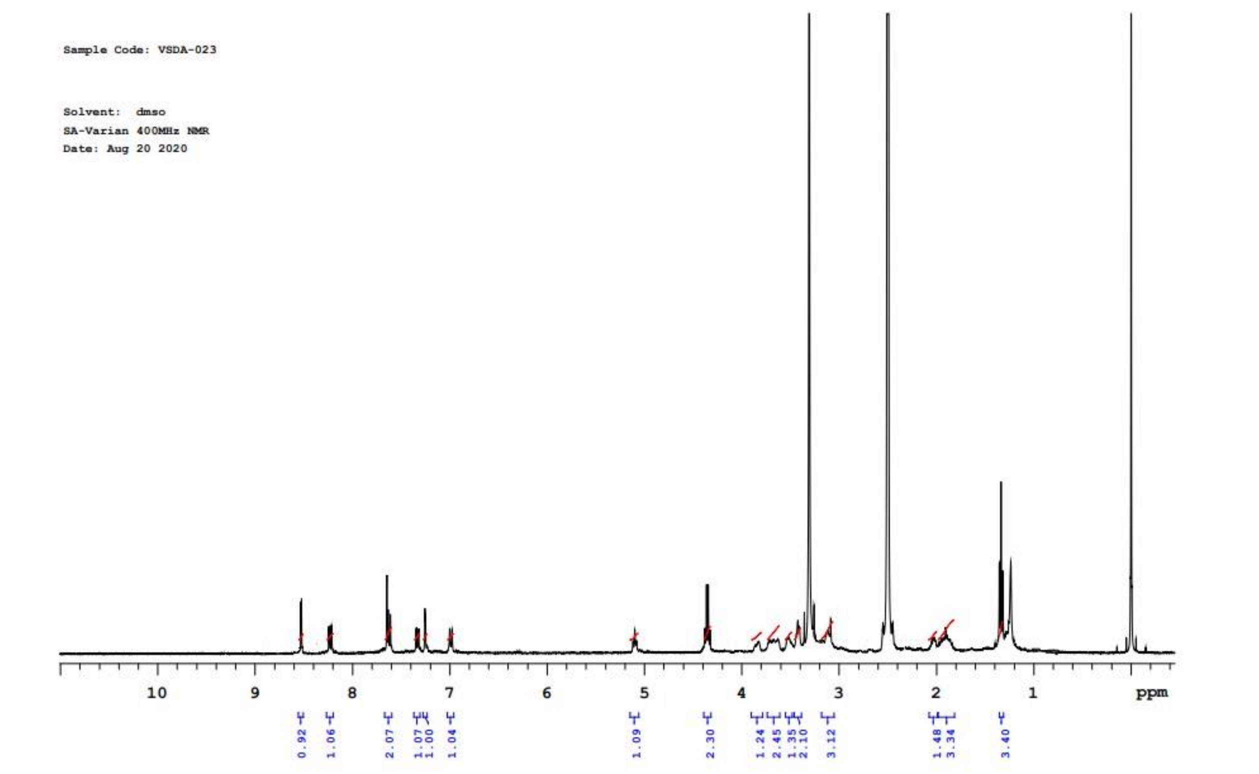

The 1H NMR of Compound 4i

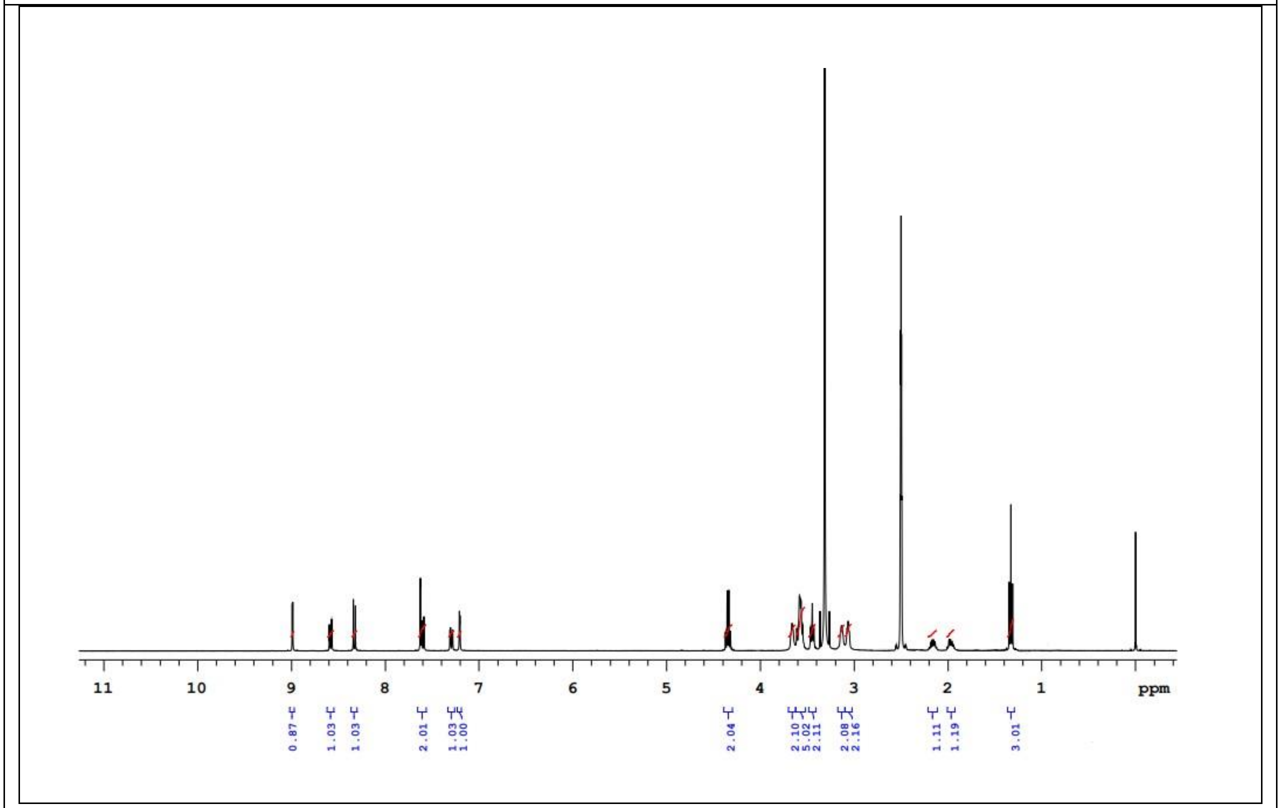

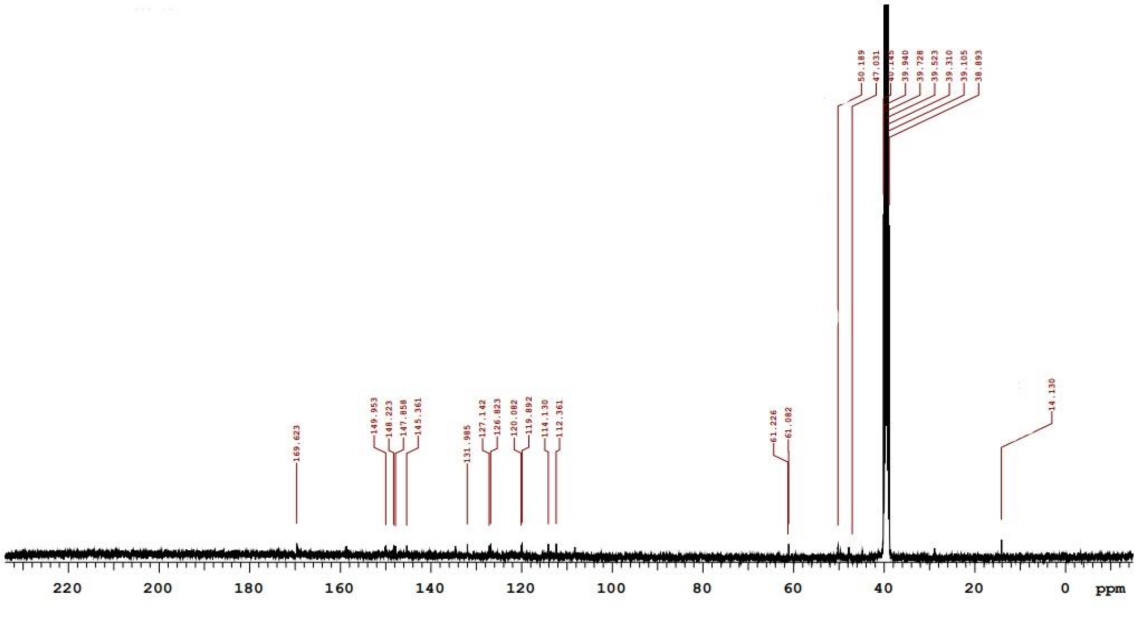

The 1H NMR of Compound 4j

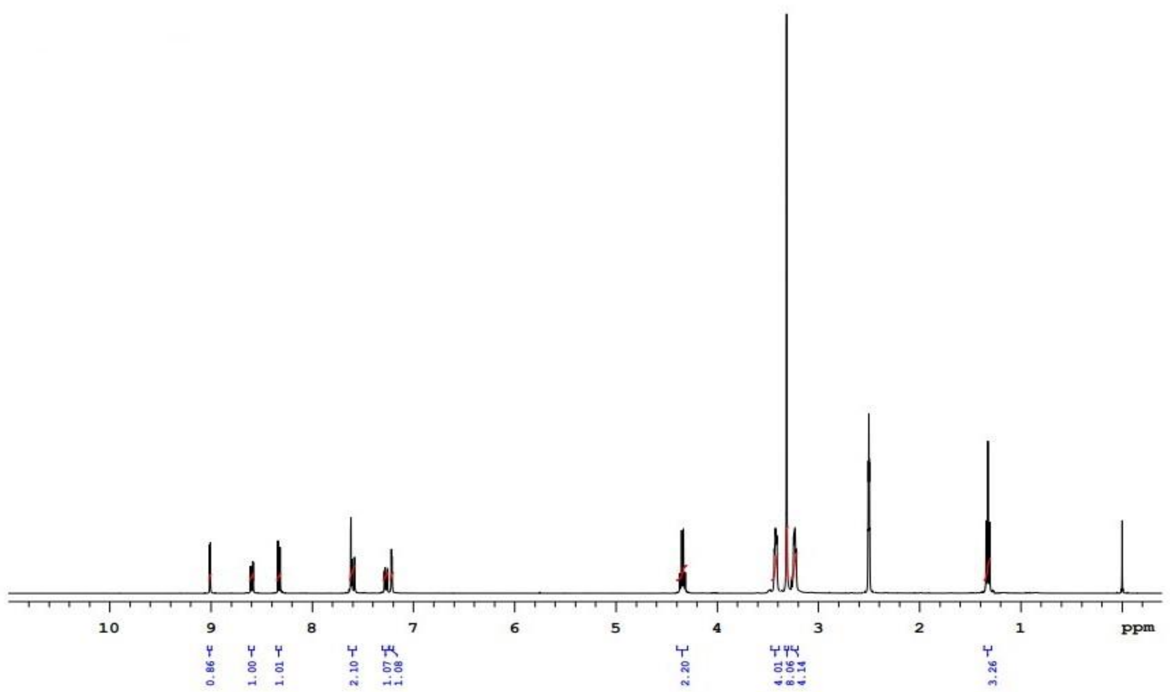

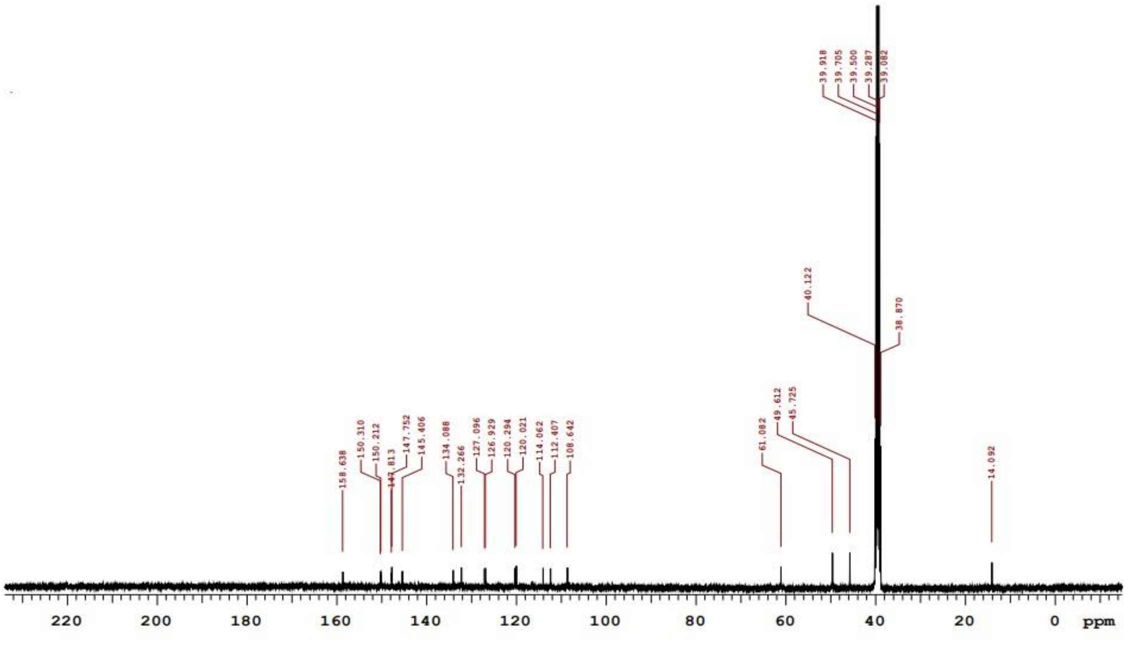

The 1H NMR of Compound 4k

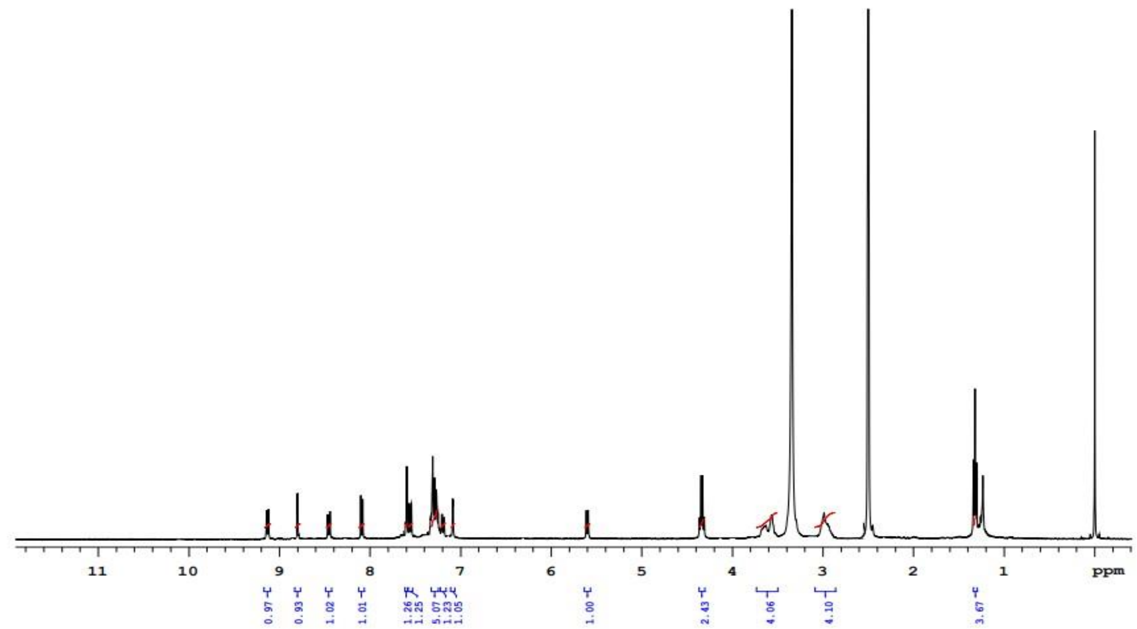

The 1H, 13C NMR and LC-MS Spectra of Compound 4m

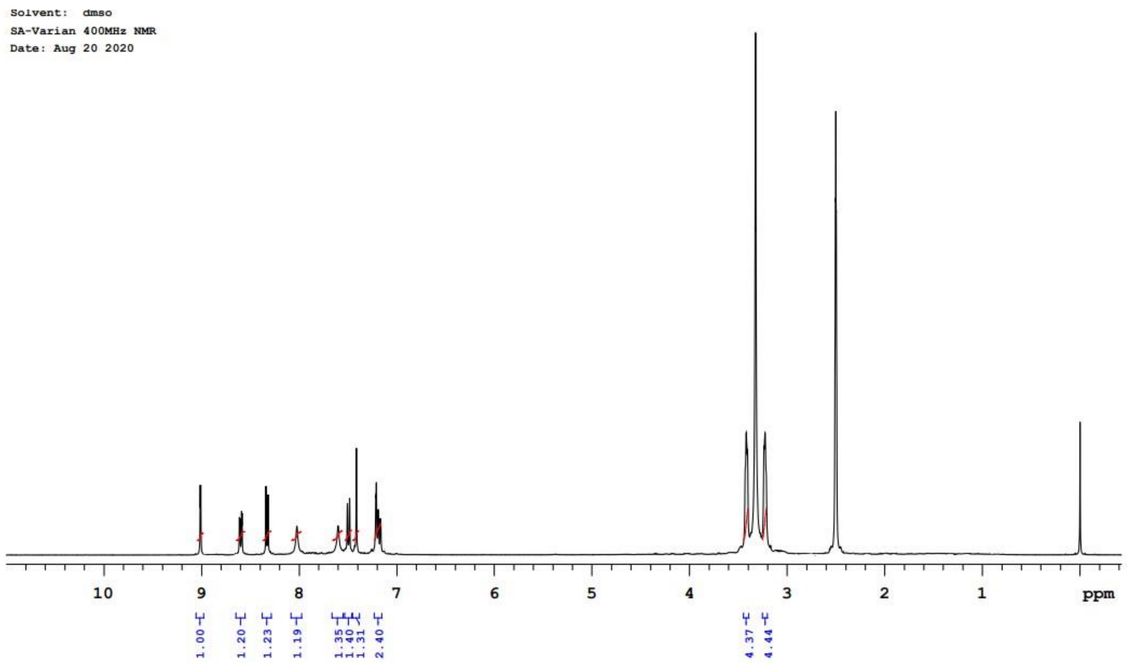

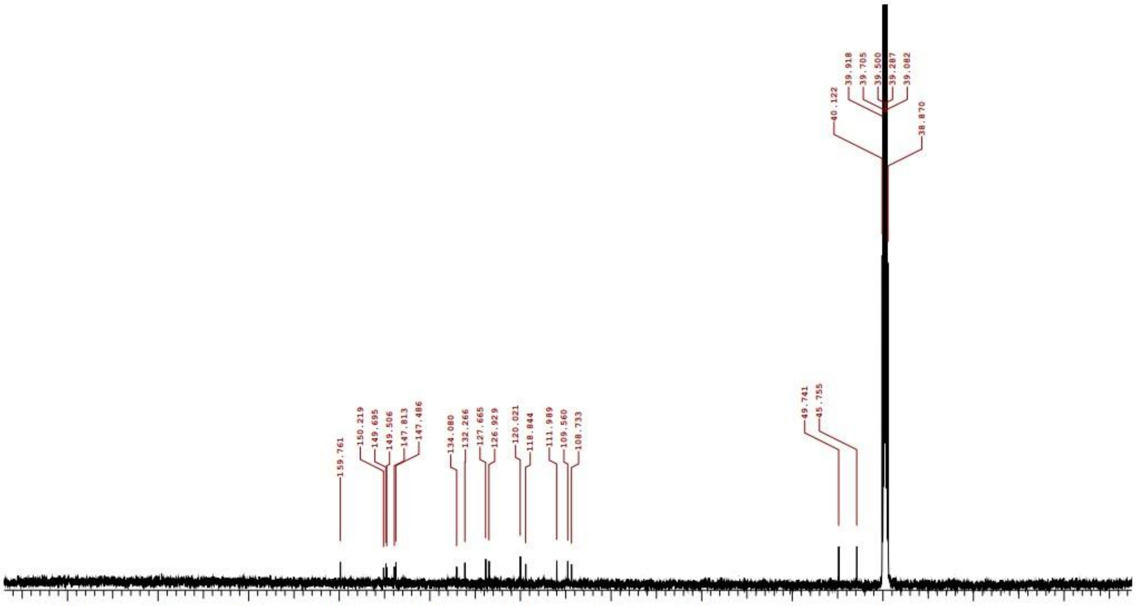

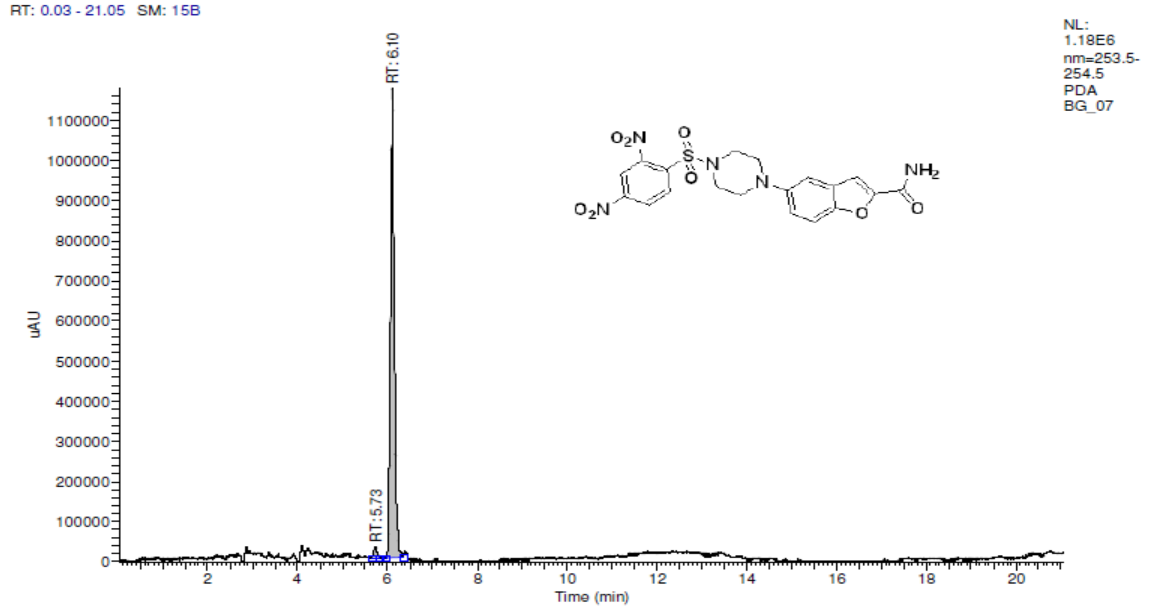

S.No.	Apex RT	Area	%Area
1	5.73	146648	2.21
2	6.10	6502112	97.79

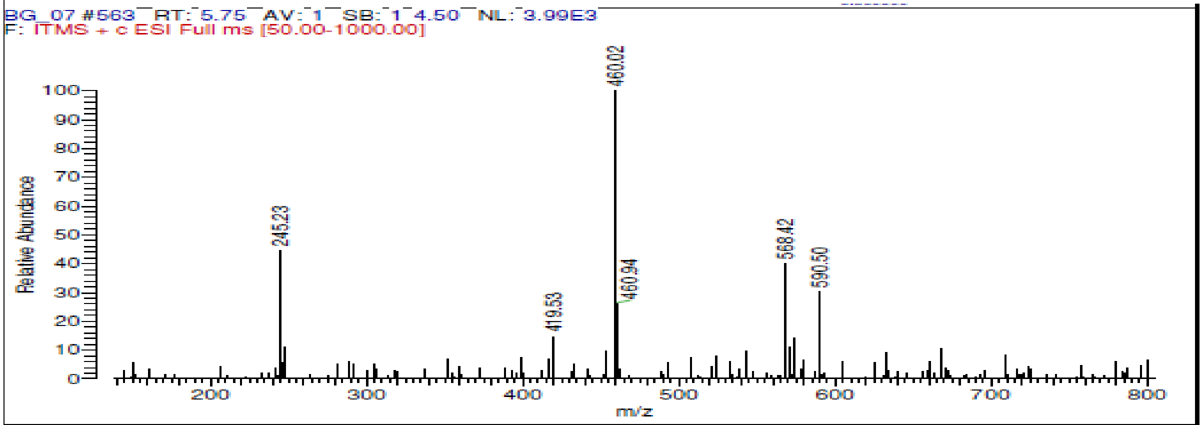

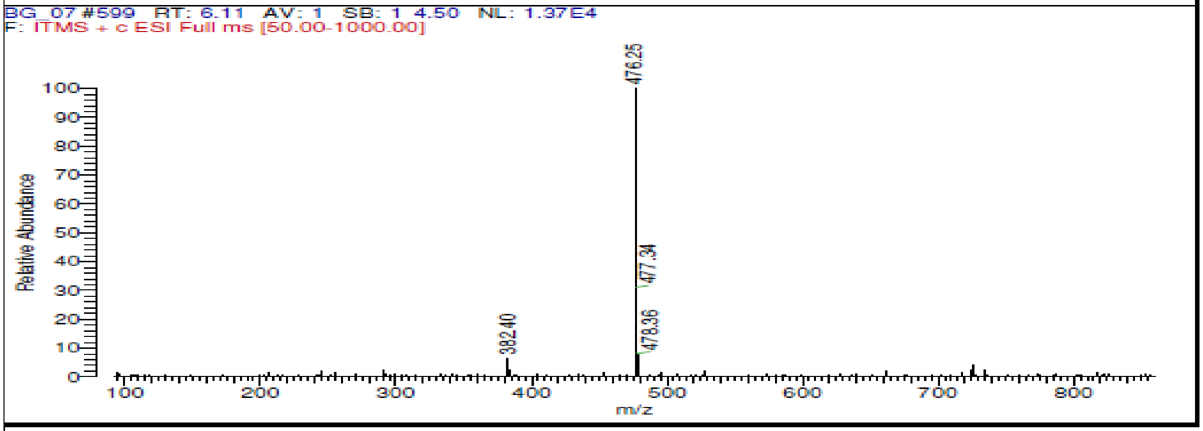

The 1H, 13C NMR spectra of Compound 4n

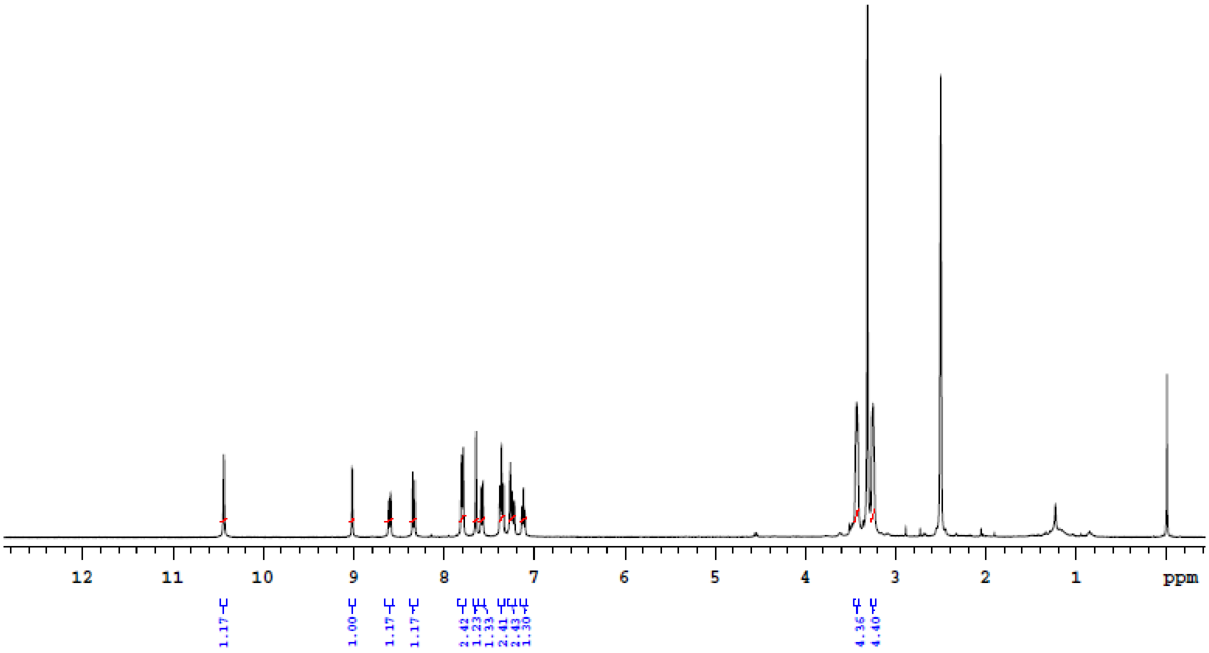

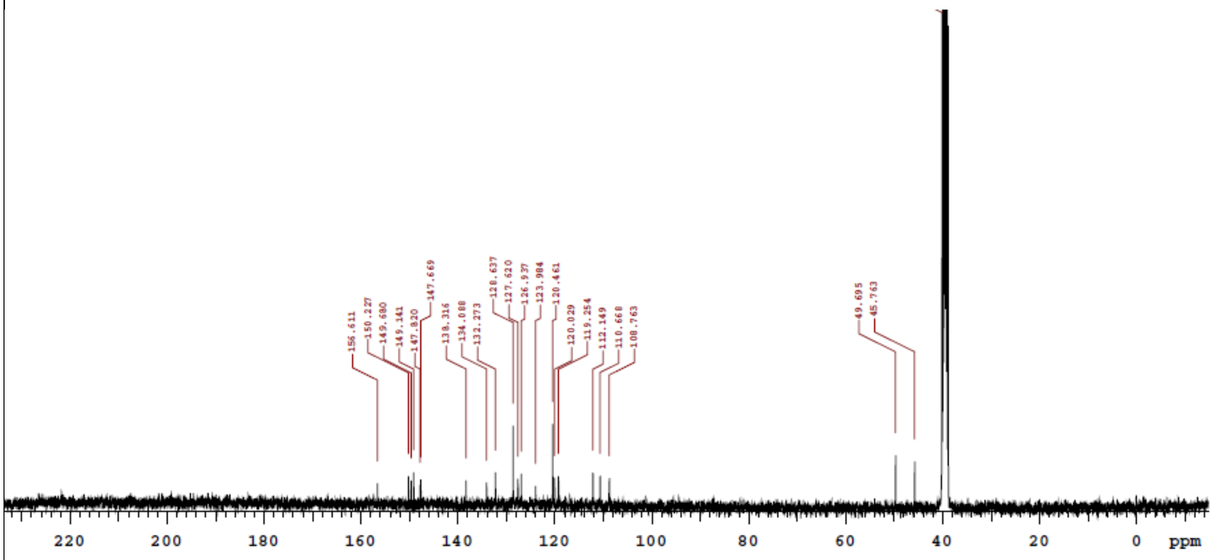

The 1H, 13C NMR spectra of Compound 4o

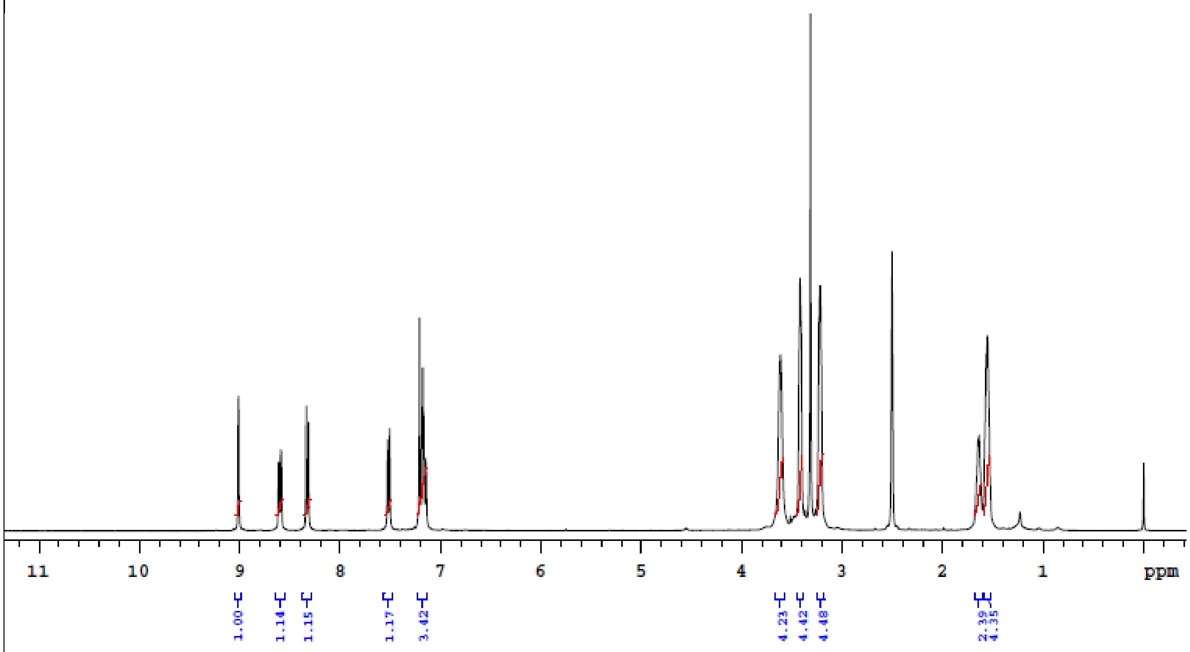

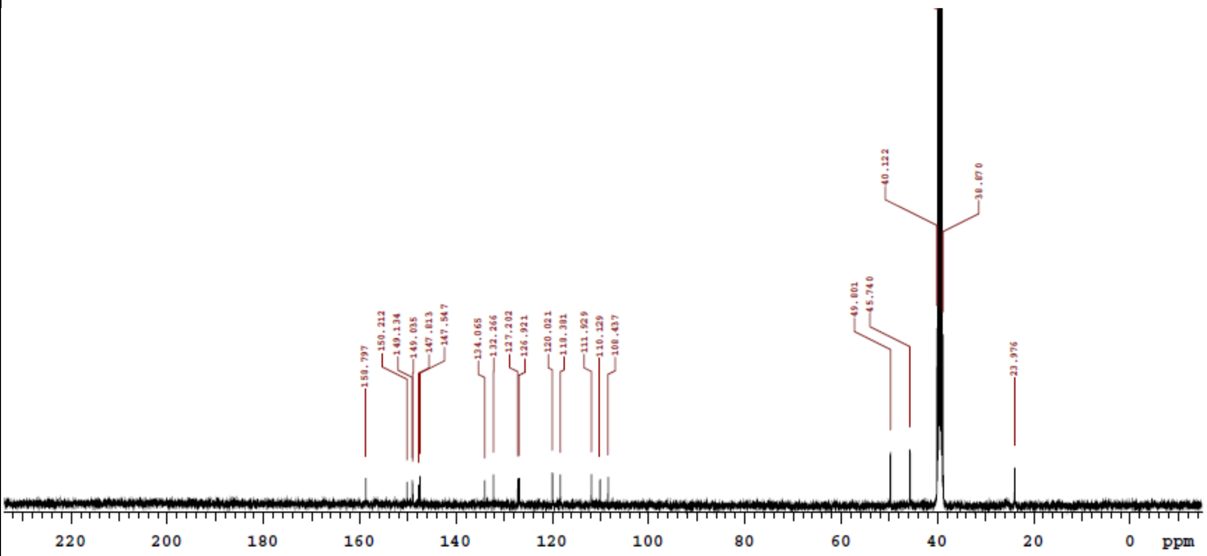

The 1H, 13C NMR spectra of Compound 4p

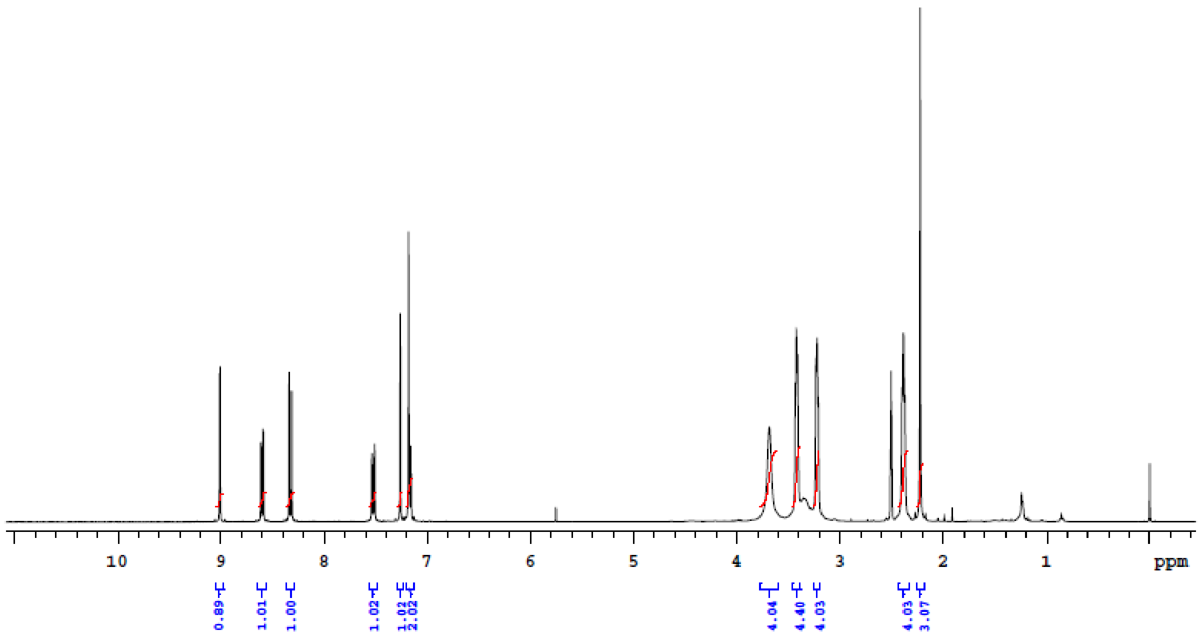

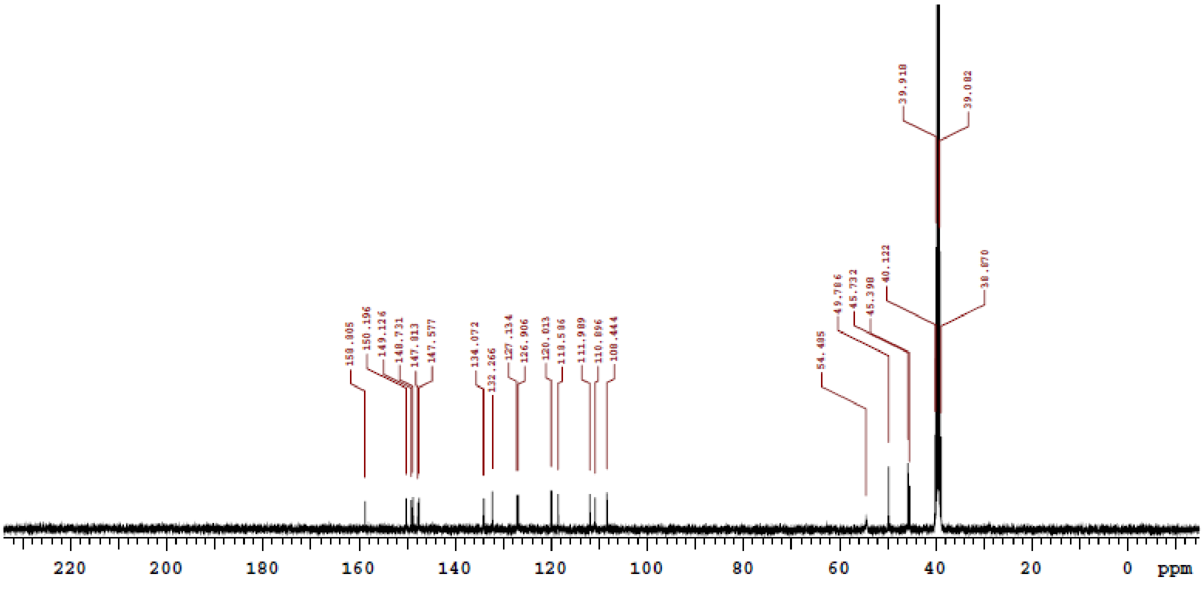

3. References

1. Malapati P, Krishna VS, Nallangi R. et al. Identification and development of benzoxazole derivatives as novel bacterial glutamate racemase inhibitors. Eur. J. Med. Chem 2018;145:23-34.
2. Collins LA, Franzblau SG, et al. Microplate alamar blue assay versus BACTEC 460 system for high-throughput screening of compounds against Mycobacterium tuberculosis and Mycobacterium avium. Antimicrob Agents Chemother 1997;41(5):1004-9.
3. Gerlier D, Thomasset N, et al. Use of MTT colorimetric assay to measure cell activation. Immunol. Methods 1986; 94(1-2): 57-63.
4. Burits M, Bucar F, et al. Antioxidant activity of Nigella sativa essential oil. Phytother Res 2000;14(5):323-8.
5. Wang F, Langley R, Gulten G, et al. Mechanism of thioamide drug action against tuberculosis and leprosy. J. Exp. Med. 2007;204(1):73-8.
6. www.rcsb.org
7. www.vlifesciences.com
8. https://discover.3ds.com/discovery-studio-visualizer-download.
9. https://www.3ds.com/products-services/biovia/references/
